# What Causes Death in Esophageal Cancer Patients Other Than the Cancer Itself: A Large Population-Based Analysis

**DOI:** 10.7150/jca.78004

**Published:** 2022-10-17

**Authors:** Xueer Zheng, Anlai Zhang, Yao Xiao, Kaibo Guo, Leitao Sun, Shanming Ruan, Fang Fang

**Affiliations:** 1 The First School of Clinical Medicine, Zhejiang Chinese Medical University, Hangzhou, Zhejiang, P. R. China.; 2 Department of medical oncology, The First Affiliated Hospital of Zhejiang Chinese Medical University (Zhejiang Provincial Hospital of Traditional Chinese Medicine), Hangzhou, Zhejiang, P. R. China.; 3 Department of Science and Education, Quzhou Hospital of Traditional Chinese Medicine, Quzhou, Zhejiang, P. R. China.; 4 Department of Science and Education, Quzhou TCM Hospital at the Junction of Four Provinces Affiliated to Zhejiang Chinese Medical University, Quzhou, Zhejiang, P. R. China.

**Keywords:** esophageal cancer, noncancer causes of death, standardized mortality ratio, cardiovascular disease, chronic obstructive pulmonary disease, septicemia, suicide and self-injury

## Abstract

**Background:** Researches on noncancer causes of death in patients with esophageal cancer (EC) are not in-depth. The objective of this paper is to broadly and deeply explore the causes of death in patients with EC, especially noncancer causes.

**Methods:** Information about the demographics, tumor-related characteristics, and causes of death of patients with EC who met the inclusion criteria were extracted from the Surveillance, Epidemiology, and End Results (SEER) database. Calculated standardized mortality ratio (SMR) for all causes of death at different follow-up times and performed subgroup analyses.

**Results:** In total, 63,560 patients with EC were retrieved from the public database. And 52,503 died during the follow-up period. Most deaths were due to EC itself within 5 years after diagnosis, but over 10 years, 59% EC patients died from noncancer causes. Cardiovascular disease was the major noncancer cause of death in patients with EC, accounting for 43%. Suicide and self-injury (2%) of EC patients should not be ignored. During the 1-year follow-up period, patients with EC had statistically highest risk of death from septicemia (SMR: 7.61; 95% CI: 6.38-9.00). Within more than 10 years after EC diagnosis, more and more patients died from chronic obstructive pulmonary disease (SMR: 2.38; 95% CI: 1.79-3.10).

**Conclusions:** Although most patients with EC still died from the cancer itself, the role of noncancer causes of death should not be underestimated. These prompt clinicians to pay more attention to the risk of death caused by these noncancer causes, which can provide relevant measures in advance to intervene.

## Introduction

Esophageal cancer (EC) is the sixth and fourth leading causes in digestive system cancer morbidity and mortality, with about 20,640 new estimated cases and 16,410 estimated deaths in 2022, globally 1. In China, large urban-rural disparities had seen in morbidity and mortality, with high indicators in rural areas during 2005 to 2015 2. Thanks to the progressive treatment mode in EC, the incidence and mortality of EC have been decreasing year by year 1, 3, 4.

Previous studies focused more on EC-related risk factors and cancer-related causes of death 5, 6, some articles also explored noncancer-related causes of death. For example, EC patients would also face some other causes of death in Sweden, where ischemic heart disease, cerebrovascular disease, and respiratory diseases were considerable 7. With the prolongation of survival time, it is necessary for us to comprehensively research the causes of death in EC patients, especially noncancer causes.

In this article, we used standardized mortality ratio (SMR) to explore the association of demographically relevant and tumor-related characteristics with varied noncancer causes of death in EC patients. Through this study with the largest scale and longest follow-up time, we provide a novel perspective for a comprehensive understanding of the causes of death in EC patients, which will provide better relevant early interventions to pursue longer survival time and higher quality of life.

## Materials and methods

### Data Resources and Subjects

We extracted information from the Surveillance, Epidemiology, and End Results (SEER) program. Because this database is a large public database, there is no need to apply for ethical approval. We used SEER*Stat software 8.3.9.2 to access the SEER 18 registries (2020 submission). The subjects of this study were to research noncancer causes of death in EC patients and assess the differences in each cause of death compared to the general population at the same time.

### Study Cohort

A total of 63,560 eligible patients from 2000 to 2018 were included. These patients all had histologic confirmation and EC was their first primary malignancies. Given the subjects of this study, we excluded patients with incomplete information, such as age, race, disease stage, tumor grade, treatment, survival status, survival time, and reasons of death.

### Variable Declaration

We gained total seven demographically relevant and tumor-related characteristics. The age of EC patients was categorized into ≤49, 50-64, and ≥65 years. The race was classified into white, black, Asian or Pacific, American Indian/Alaska Native. For disease stage, we used SEER stage and divided EC into localized, regional, and distant. The degree of tumor differentiation was divided into four grades: Grade I (well differentiation), Grade II (moderately differentiation), Grade III (poorly differentiation), and Grade IV (undifferentiation, anaplastic). Treatment options were cancer-directed surgery, radiation and chemotherapy.

We classified the causes of death in patients with EC into all causes, all malignant cancer causes, esophageal cancer deaths, and 24 noncancer causes of death. These 24 noncancer causes of death covered diseases of respiratory system, cardiovascular system, digestive system, urinary system, some unexpected and adverse events. The following are examples of 24 noncancer causes of death: tuberculosis, diseases of heart, atherosclerosis, pneumonia and influenza, stomach and duodenal ulcers, chronic liver disease and cirrhosis, nephritis, nephrotic syndrome and nephrosis, accidents and adverse effects, suicide and self-inflicted injury. Causes of death has been based on the International Statistical Classification of Diseases and Related Health Problems, 10th Revision (World Health Organization), For example, heart disease includes codes I00-I02 (Acute rheumatic fever), I05-I09 (Chronic rheumatic heart diseases), I11 (Hypertensive heart disease), I13 (Hypertensive heart and renal disease), I20-I25 (Ischemic heart diseases), I26-I28 (Pulmonary heart disease and diseases of pulmonary circulation), I30-I32 (Diseases of pericarpdium), I33 (Acute and subacute endocarditis), I34-I39 (Nonrheumatic valve disorders), I40-I41 (Myocarditis), I42-I43 (Cardiomyopathy), I44-I45 (Conduction disorders), I46 (Cardiac arrest), I47-I49 (Arrythmias), I50 (Heart failure), I51 (Complications and ill-defined descriptions of heart disease).

Firstly, we described the percentage of people who died from different causes at different follow-up times (<1 year, 1-5 years, 5-10 years, and >10 years after EC diagnosis). Secondly, calculated the SMR for various causes of death in patients with EC. SMR can show differences in the risk of dying from the same cause between a specific population and the general population. SMR is the observed-to-expected ratio, that is, the number of people diagnosed with EC who died from a specific cause between 2000 and 2018, compared to the number of people in the general population who are likely to die from the same cause with similar demographic factors, in this article.

### Statistical Analysis

With SEER*Stat software 8.3.9.2, we derived SMR for different causes of death and calculated with its 95% confidence intervals (CI). Furthermore, we performed corresponding subgroup analyses. If the number of deaths observed in EC patients was greater than the expected number of deaths in the demographically similar population, the risk of that specific cause of death was considered to be significantly increased, which P-value was < 0.05 (two sided).

## Results

### Demographic and Clinical Features

63,560 patients diagnosed with EC in 2000-2018 were included in this paper. Most of them were aged >64 years old (64.41%), white (83.79%), male (77.12%) and diagnosed with advanced (55.12%), moderately or poorly differentiated (68.26%) EC. Cancer-directed surgery was not recommended for most EC patients, but more than half of them chosen radiation or chemotherapy. A total of 52,503 death cases occurred during the follow-up time and the average age of death was 69.70 years old. The number of deaths and follow-up time of EC patients showed an obvious inverse trend, which proved the poor prognosis and aggressiveness of EC: 32,923 deaths (62.7%) occurred during less than a year after EC diagnosis with the average age of 69.65 years old. 16,731 deaths (31.9%) occurred in 1-5 years follow-up time with the average age of 68.78 years old. 2,134 deaths (4.1%) occurred within 5-10 years after EC diagnosis with the average age of 74.68 years old. 715 deaths (1.4%) occurred during more than 10 years after EC diagnosis with the average age of 78.85 years old. The relevant data are recorded in detail in **Table 1**.

### Noncancer Causes of Death less than a year after EC Diagnosis

**Figure 1:**
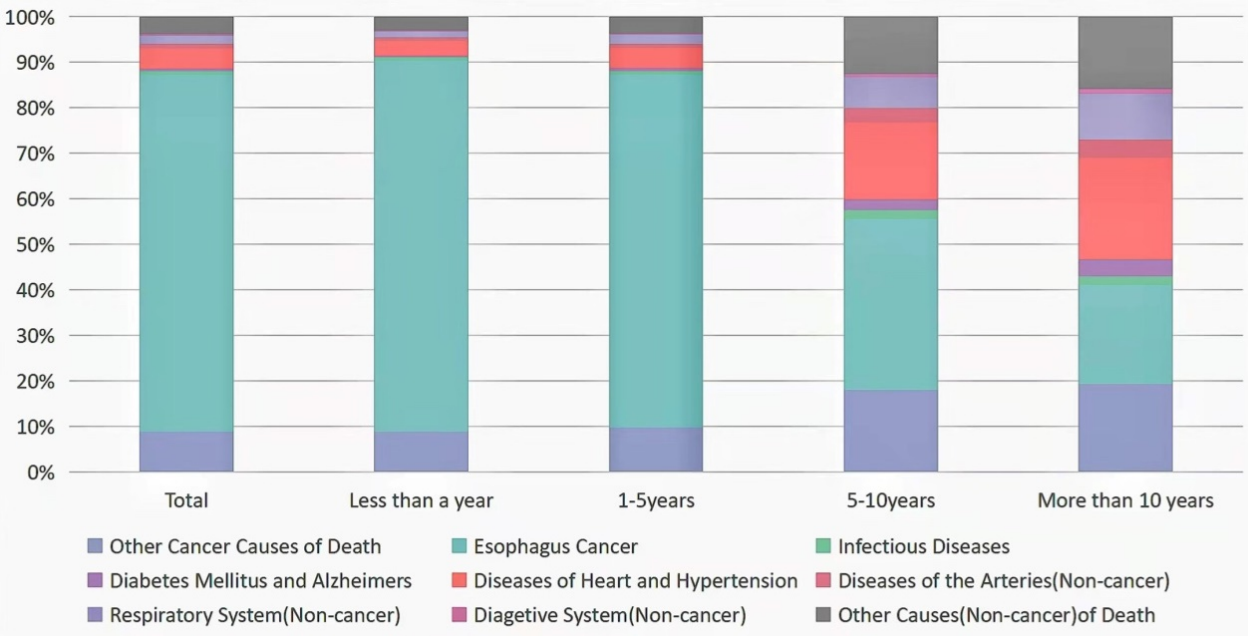
Causes of death in each latency period after esophageal cancer diagnosis.

There were 32,923 death cases reported during 1-year follow-up period after EC diagnosis. 29,696 died from malignant tumors, including 26,873 died from EC. Among deaths from noncancer causes, the largest proportion in this latency period (<1 year after EC diagnosis) was disease of heart (1,081 deaths; 3%), followed by chronic obstructive pulmonary disease (COPD) (273 deaths; 0.8%), cerebrovascular diseases (180 deaths; 0.05%), and septicemia (136 deaths; 0.04%) (**Table 2, Fig. 1**). EC patients were more prone to commit suicide and self-inflicted injury in this time with highest SMR of 9.16 (95% CI, 7.36-11.27). In the meantime, we also discovered that patients with EC had a higher risk of death from septicemia (SMR = 7.61, 95% CI = 6.38-9.00, p < 0.05) and other infectious diseases (SMR = 7.50, 95% CI = 5.94-9.35, p < 0.05) (**Table 2**).

In the subgroup analysis, we saw almost the same trend as the total population, with disease of heart as the leading cause of death (for details of each subgroup analysis, see attached **Supplementary Tables 1-20**), followed by COPD (**Supplementary Tables 2-6, 10-19**).

Patients after EC diagnosis seemed to have a higher risk of septicemia, pneumonia, influenza and other infectious diseases, regardless of age, sex and race (except American Indian/Alaska Native). Interestingly, we found that suicide and self-inflicted injury were more common in male patients within the first year after EC diagnosis, which was different from traditional view (**Supplementary Tables 4,5**). White, black, Asian or Pacific islander people with EC had a statistically higher rate of death from noncancer causes than the general people, while American Indian or Alaska Native did not (see **Supplementary Tables 6-9**).

In digestive diseases, patients with localized EC diagnosis had a significantly increase of death from stomach and duodenal ulcers, while regional or distant EC patients had a significantly increase of death from chronic liver disease and cirrhosis (**Supplementary Tables 10-12**). Grade I to III EC patients were more likely died from cerebrovascular diseases but Grade IV EC patients were more likely died from atherosclerosis (**Supplementary Tables 13-16**). The risk of death from infectious diseases increased compared to the general population, with or without surgery (see **Supplementary Tables 17-18**).

### Noncancer Causes of Death within 1-5 years after EC Diagnosis

In total, 16,731 people diagnosed with EC died within 1 to 5 years, of whom 14,558 died from all malignant cancer causes, and 12,980 died from EC. Among deaths from noncancer causes, the largest proportion in this latency period (1-5 years after EC diagnosis) was disease of heart (755 deaths; 4.5%), followed by COPD (243 deaths; 1.5%), and cerebrovascular diseases (106 deaths; 0.06%). All statistically significant SMR were increased, except for Alzheimer's disease, which had a lower risk of death compared to the general population. At this period, infectious diseases (septicemia, pneumonia and influenza) and unexpected events (accidents, adverse effects, suicide and self-inflicted injury) remained an increased risk factor for death in EC patients, but the risk of death was reduced compared to within 1-year risk of death (**Table 2, Fig. 1**).

In subgroup analyses based on demographic and tumor-related features, diseases of heart were the leading non-cancer cause of death (**Supplementary Tables 2, 4-8, 10-20**). Further, we found that mortality from diseases of heart decreased within 1-5 years compared to within 1 year.

In demographic-related subgroups, EC patients aging 0-49 years had a statistically higher risk of death from pneumonia and influenza, and older than 64 years patients were more likely to die from septicemia (**Supplementary Tables 1-3**). Female patients had a statistically increased death rate of cardiac or respiratory diseases at this time (**Supplementary Table 5**), additionally, male patients also had a statistically higher risk of death from digestive system diseases including the above-mentioned diseases (**Supplementary Table 4**). Both white and black patients were more likely to die from cardiovascular, respiratory, digestive, urinary system diseases compared to general US population. Conversely, Native American/Alaska Native patients did not have an increased risk of non-cancer causes (**Supplementary Tables 6,7,9**).

In tumor-related subgroups, patients with localized EC diagnosis had a significant higher risk of death from nephritis, nephrotic syndrome and nephrosis, compared with the general US population, with SMR of 1.92 (95% CI, 1.17-2.96). Receiving radiotherapy or chemotherapy were associated with a significant higher risk of death from aortic aneurysm and dissection at this period (**Supplementary Tables 19,20**).

### Noncancer Causes of Death within 5-10 years after EC Diagnosis

In total, there were 2,134 death cases reported within 5-10 years after EC diagnosis. 1,170 died from malignant tumors, including 800 died from EC. Among deaths from noncancer causes, the largest proportion in this latency period (5-10 years after EC diagnosis) was disease of heart (349 deaths; 16.35%), followed by COPD (107 deaths; 5.01%), cerebrovascular diseases (58 deaths; 2.72%) (**Table 2, Fig. 1**). EC patients had a statistically increase of death from septicemia, pneumonia and influenza within 5-10 years after cancer diagnosis, with SMR of 2.80 (95% CI, 1.90-3.97) and 2.13 (95% CI, 1.51-2.93), respectively (**Table 2**). The most potential cause of death found in specific subgroups was diseases of heart, which was the same as the total population (**Supplementary Tables 2, 4-20**).

EC patients older than 64 were more likely to die from septicemia, regardless of gender (see **Supplementary Tables 3-5**). White EC patients had a statistically higher risk of death from tuberculosis, with SMR of 9.98 (**Supplementary Table 6**). Patients who were diagnosed with Grade I EC, and patients who underwent cancer-directed surgery, radiotherapy or chemotherapy associated with a statistically higher risk of death from pneumonia and influenza during 5-10 years after diagnosis (**Supplementary Tables 13, 17, 19, 20**).

### Noncancer Causes of Death more than 10 years after EC Diagnosis

A total of 715 patients with EC died more than 10 years follow-up, of whom 293 died from malignant cancer causes, and 156 died from EC. Among deaths from noncancer causes, the largest proportion in this latency period (>10 years after EC diagnosis) was disease of heart (154 deaths; 21.54%), followed by COPD (55 deaths; 7.69%), cerebrovascular diseases (22 deaths; 3.08%), and Alzheimer's disease (19 deaths; 2.66%) (**Table 2, Fig. 1**). EC patients were more prone to die from septicemia in this time with SMR of 2.29 (95% CI, 1.18-4.01). Disease of heart was still the most common non-cancer cause in subgroups analysis at the same period (**Supplementary Tables 1, 2, 4-8, 10-20**).

Interesting, we found that older than 64 years patients with EC most died from atherosclerosis (142 deaths) and nephritis, nephrotic syndrome and nephrosis (52 deaths) at this latency period (**Supplementary Table 3**). White and black male EC patients who receiving chemotherapy were more prone to die from septicemia, pneumonia and influenza (**Supplementary Tables 4, 6, 7, 20**). Patients who was diagnosed with localized EC had a statistically higher risk of death from Alzheimer's disease, with SMR of 2.76 (95% CI, 1.38-4.93) (see **Supplementary Table 10**), while Grade III patients were more prone to die from hypertension without heart disease (**Supplementary Table 15**).

### Risk Overview for Relatively Controllable Causes of Death after EC Diagnosis

**Figure 2:**
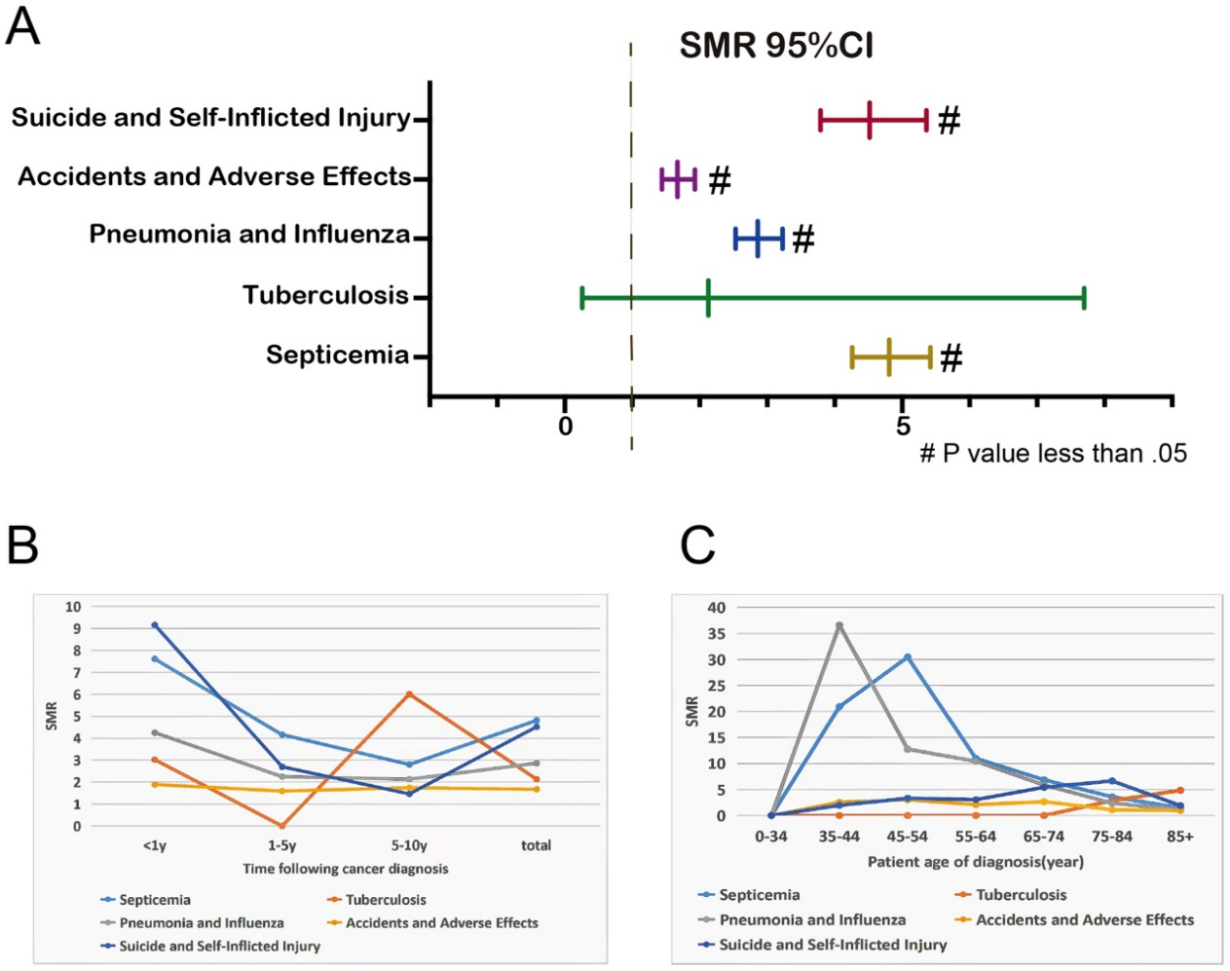
SMR of infectious diseases and unexpected events at total **(A)**; SMR of infectious diseases and unexpected events at different latency periods **(B)**; SMRs of infectious diseases and unexpected events at different age **(C)**.

Considering the high aggressiveness and poor prognosis of EC itself, we focus more on non-cancer causes of death that can be detected early and given corresponding interventions, such as some infectious diseases (septicemia, tuberculosis, pneumonia and influenza) and unanticipated events (accidents and adverse effects, suicide and self-inflicted injury). Overall, patients with EC had a higher risk of death from these 5 diseases than the general US population (**Figure 2A**). Except for the 1-5 years follow-up period, tuberculosis mortality rate was lower than that of the general population, the mortality rates of these 5 diseases in other follow-up years were higher than that of the general population (**Figure 2B**). Younger EC patients were more likely to have life-threatening because of septicemia, pneumonia and influenza (**Figure 2C**).

## Discussion

Esophageal cancer is one of the most lethal malignancies. According to the World Bank's Human Development Index (HDI), the incidence and morbidity of cancer increased as the socio-demographic index (SDI) increased 8. The cancer burden of EC will increase in the world. On the other hand, with the improvement of treatment methods 9-12, the survival rate and survival time of EC patients also continue to improve 13. Therefore, as the survival period of EC patients prolongs, the cause of their death is not only due to the cancer itself, but also the influence of non-tumor causes. However, deaths caused by non-tumor causes have not received extensive attention from researchers. This article is an extensive and profound study of non-tumor causes of death in patients with EC.

In our paper, most deaths were due to EC itself within 5 years after diagnosis. But over time, more and more patients died from non-tumor causes. When we followed up over 10 years, more than half of EC patients died from non-tumor causes (**Figure 1**). The main non-cancer causes of death in EC patients were cardiovascular, respiratory and infectious diseases.

With aggressive treatment in the initial few years (usually within 1 year) after diagnosis, patients with EC experienced treatment-related cardiotoxicity such as pericarditis, cardiomyopathy, valve abnormalities, arrhythmias, and so on. Radiotherapy plays an important role in the treatment of patients with esophageal cancer. Due to the special location of the heart and esophagus, radiation therapy for EC patients also causes accidental exposure to the heart 14. The reactive oxygen species formed by radiation damages tumor cells on the one hand, and also causes inflammation and destruction of cardiovascular blood vessels on the other hand 15, 16. Autopsy found that heart valves experienced fibrotic changes when exposed to radiation, but the pathophysiology is still not well illustrated 17, 18, Radiation-induced heart disease (RIHD) initially presents with pericardial disease such as pericardial effusion and pericarditis, and eventually progresses to restrictive heart disease, myocardial infarction, and heart failure 19, 20. Although proton beam therapy, which is advanced in recent years, has reduced cardiotoxicity to some extent, the burden remains 21.

Antimetabolites, microtubule inhibitors, platinum-based chemotherapy drugs are the cornerstone of chemotherapy regimens for EC. 5-Fluorouracil and capecitabine are common antimetabolites that damage proliferating cells during the S phase of mitosis by interfering with DNA/RNA growth. It had been reported that the cardiotoxicity of 5-fluorouracil manifested as myocardial ischemia, angina pectoris and chest pain, and its incidence was approximately between 1% and 7.6% 22, 23. Capecitabine also exhibits similar cardiotoxicity, the mechanism might be related to the formation of thrombus after endothelial injury, oxidative stress damaged cells, coronary artery spasm and myocardial ischemia 24. Microtubule inhibitors are widely known as taxanes such as paclitaxel and docetaxel. Paclitaxel inhibits cell mitosis by disrupting microtubule function 24, their most common effect on the heart was arrhythmia, which could not only cause bradyarrhythmias such as bradycardia, heart block 25, but also supraventricular arrhythmias such as atrial fibrillation, atrial flutter, and atrial tachycardia 26. The most worrying consequence of platinum-based chemotherapy is vascular toxicity. Platinum had been reported to be detectable in serum for several years after completion of cisplatin therapy 27. The mechanism of cardiotoxicity caused by platinum-based chemotherapy drugs might be related to mitochondrial abnormalities, endoplasmic reticulum stress, then inhibited cardiomyocyte contraction 28. In this article's subgroup analysis, chemotherapy-experienced EC patients indeed had a higher risk of cardiac death than the US general population. Targeted drug trastuzumab is used in patients with HER2-positive esophageal cancer. Trastuzumab was originally used to treat breast cancer and was found to improve breast cancer outcomes while increasing the risk of cardiac insufficiency, but this risk was resolved once treatment was stopped 29. Drugs targeting HER2 often cause asymptomatic cardiac insufficiency and symptomatic heart failure 30. HER/ERBB biological overlap between tumor and cardiovascular system led to potential cardiovascular impact 24. Immune checkpoint inhibitors (ICIs) have changed the treatment landscape for many cancers. Phase III CheckMate 649 study transformed the first-line treatment of esophageal adenocarcinoma with encouraging results, adding nivolumab to standard chemotherapy could prolong overall survival (HR 0.71; 98.4% CI 0.59-0.86; p < 0.0001) and progression-free survival (HR 0.68; 98% CI 0.56-0.81; p < 0.0001) 31. Cardiovascular toxicities of ICIs included myocarditis, pericarditis, arrhythmias, and atherosclerosis 32-35. The role of T cells in the development of cardiotoxicity was definitely clear 36.

The incidence of respiratory diseases is also high in cancer patients. The occurrence of pneumonia was not only associated with neutropenia in tumor patients 37, but also with immune-related pneumonia caused by ICIs 38. The risk of developing esophageal cancer was also significantly higher in COPD patients (the standardized incidence ratio: 1.35, 95% CI 1.08-1.67, p = 0.010) 39. This article also drawn the same conclusion that patients with EC were at high risk of die from respiratory disease, either treated with chemoradiotherapy or surgery.

Blood routine trilineage cell reduction is a difficult problem in the process of tumor treatment 40, 41. Neutropenia made sepsis more likely to happen, this could be fatal in cancer patients 42. A study shown that despite aggressive care and treatment, in-hospital mortality from sepsis was higher in cancer patients than in those without cancer 43. Tumor invasion and metastasis make cancer patients immunocompromised and more susceptible to opportunistic infections such as tuberculosis, which can lead to fatal consequences. Cancer patients had a higher suicide rate than the general population, with one study reporting that EC patients were five times more likely to commit suicide than the general population 44. Elderly white men with EC who had not undergone surgery or chemotherapy were at high risk for suicide 45. Esophageal cancer might be an important risk factor for anxiety and depressive disorders 46, but one study showed that people with EC anxiety were more likely to receive anti-cancer treatment than those without anxiety 47. This suggested that the involvement of psychiatrists and other mental health professionals may be a key component of cancer care and treatment in these high-risk patient subgroups.

To our knowledge, this study is the largest sample size and longest follow-up time study about non-cancer deaths in patients with EC. Through different follow-up periods and careful subgroup analysis, we deeply and comprehensively explored non-cancer causes in EC patients. However, as a retrospective study, there were inevitably some biases. We reduced selection bias and confounding bias to the greatest extent by tightly controlling screening criteria and adjusting for covariates. With the continuous advancement of medicine, the SEER database has not yet updated some key patient information that might affect treatment, such as gene mutations. In the future, further researches on the impact of relevant factors on the death of patients with EC is required.

In conclusion, more and more patients with EC died from non-tumor causes with the prolongation of survival time. The main non-tumor causes of death in EC patients are cardiovascular diseases, respiratory diseases and infectious diseases. In addition, deaths from unexpected events such as suicide and self-injury cannot be ignored. Timely and adequate anti-infective treatment and psychological intervention are important. These prompt clinicians to pay more attention to the risk of death caused by these noncancer causes, which can provide relevant measures in advance to intervene.

## Supplementary Material

Supplementary tables.

## Figures and Tables

**Table 1:** Demographic and clinical features of all EC patients and those who died according to the time of death after diagnosis

Timing of death after diagnosis		All Death		<1 Year		1-5 Years		5-10 Years		>10 Years	
Characteristic	Total No. of Patients	No. of Patients (%)	Mean Age at Death, y	No. of Patients (%)	Mean Age at Death, y	No. of Patients (%)	Mean Age at Death, y	No. of Patients (%)	Mean Age at Death, y	No. of Patients (%)	Mean Age at Death, y
**Overall**	63560	52503(100)	69.70	32923(62.7)	69.65	16731(31.9)	68.78	2134(4.1)	74.68	715(1.4)	78.85
**Age at diagnosis, y**											
0-49	4445	2814(100)	44.84	1902(67.6)	44.99	873(31.0)	44.48	34(1.2)	44.99	5(0.2)	48.62
50-64	23319	15984(100)	58.74	10149(63.5)	58.63	5434(34.0)	58.85	348(2.2)	59.73	53(0.3)	61.32
>64	41065	33705(100)	76.98	20872(61.9)	77.25	10424(30.9)	76.00	1752(5.2)	78.23	657(1.9)	80.49
**Sex**											
Male	49083	40488(100)	68.65	25145(62.1)	68.42	13181(32.6)	68.09	1643(4.1)	73.79	519(1.3)	78.16
Female	14477	12015(100)	73.24	7778(64.7)	73.63	3550(29.5)	71.37	491(4.1)	77.67	196(1.6)	80.65
**Race**											
White	53256	43761(100)	70.26	27054(61.8)	70.32	14236(32.5)	69.16	1842(4.2)	74.88	629(1.4)	79.19
Black	7028	6218(100)	65.66	4237(68.1)	65.25	1715(27.6)	65.62	209(3.4)	72.02	57(0.9)	74.92
Asian or Pacific Islander	302	253(100)	65.78	172(68.0)	66.50	70(27.7)	63.24	9(3.6)	69.85	2(0.8)	73.83
American Indian/Alaska Native	2974	2271(100)	70.46	1460(64.3)	70.42	710(31.3)	69.43	74(3.3)	77.95	27(1.2)	79.54
**Disease stage**											
Localized	10004	6425(100)	74.35	3117(48.5)	74.60	2543(39.6)	73.20	631(9.8)	76.42	134(2.1)	80.72
Regional	16036	11889(100)	69.33	5898(49.6)	69.78	5308(44.6)	68.30	588(4.9)	72.96	95(0.8)	76.21
Distant	18998	17119(100)	66.53	12876(75.2)	66.83	4096(23.9)	65.40	126(0.7)	71.24	21(0.1)	76.54
**Tumor grade**											
Well differentiated: Grade I	2750	2041(100)	70.84	1035(50.7)	70.61	765(37.5)	69.59	177(8.7)	74.08	64(3.1)	80.62
Moderately differentiated: Grade II	19129	15987(100)	69.42	9053(56.6)	69.29	5889(36.8)	68.53	783(4.9)	74.74	262(1.6)	78.00
Poorly differentiated: Grade III	24254	21616(100)	69.10	14127(65.4)	69.26	6620(30.6)	67.99	655(3.0)	73.76	214(1.0)	78.34
Undifferentiated, anaplastic: Grade IV	957	850(100)	69.89	560(65.9)	69.50	242(28.5)	69.18	33(3.9)	77.31	15(1.8)	79.73
**Cancer-directed surgery**											
Yes	15894	9995(100)	67.79	3145(31.5)	66.98	5273(52.8)	66.36	1137(11.4)	72.73	440(4.4)	77.95
Not recommended	38196	34023(100)	69.80	24082(70.8)	69.60	9018(26.5)	69.54	727(2.1)	76.93	196(0.6)	80.07
**TNM stage**											
**T**											
Tis	20	19(100)	67.88	14(73.7)	68.18	4(21.1)	66.02	1(5.3)	71.17	0(0.0)	/
T1	10832	8607(100)	71.43	4913(57.1)	71.00	2990(34.7)	70.81	585(6.8)	76.35	119(1.4)	80.60
T2	3392	2668(100)	70.30	1095(41.0)	69.80	1327(49.7)	69.69	207(7.8)	75.19	39(1.5)	78.99
T3	10621	8996(100)	68.31	4281(47.6)	68.47	4229(47.0)	67.61	422(4.7)	72.72	64(0.7)	75.12
T4	5060	4764(100)	66.31	3444(72.3)	66.43	1213(25.5)	65.63	90(1.9)	69.78	17(0.4)	76.97
Tx	9969	9388(100)	70.62	7069(75.3)	70.81	2119(22.6)	69.46	166(1.8)	75.09	34(0.4)	79.56
Unknown	23666	18061(100)	69.9	12107(67.0)	69.75	4849(26.8)	68.80	663(3.7)	74.88	442(2.4)	78.92
**N**											
N0	16393	13238(100)	71.62	7361(55.6)	71.36	4840(36.6)	71.03	858(6.5)	75.55	179(1.4)	79.38
N1	17458	15548(100)	67.14	9167(59.0)	67.09	5827(37.5)	66.65	488(3.1)	72.50	66(0.4)	77.22
Nx	6043	5656(100)	71.63	4288(75.8)	71.92	1215(21.5)	69.97	125(2.2)	76.24	28(0.5)	78.13
Unknown	23666	18061(100)	69.9	12107(67.0)	69.75	4849(26.8)	68.80	663(3.7)	74.88	442(2.4)	78.92
**M**											
M0	23117	18481(100)	71.20	8958(48.5)	71.56	8005(43.3)	69.99	1278(6.9)	74.72	240(1.3)	78.97
M1	13398	12848(100)	66.19	9592(74.7)	66.50	3148(24.5)	65.05	91(0.7)	70.96	17(0.1)	77.38
Mx	3379	3113(100)	74.18	2266(72.8)	74.94	729(23.4)	71.48	102(3.3)	76.27	16(0.5)	76.61
Unknown	23666	18061(100)	69.90	12107(67.0)	69.75	4849(26.8)	68.80	663(3.7)	74.88	442(2.4)	78.92
**Histologic type**											
EAS	35052	27903(100)	69.29	16541(59.3)	69.33	9826(35.2)	68.26	1174(4.2)	74.22	362(1.3)	78.85
ESCC	20836	17814(100)	69.93	11603(65.1)	69.50	5187(29.1)	69.60	750(4.2)	75.53	274(1.5)	79.14
Others	7672	6786(100)	70.84	4779(70.4)	71.11	1718(25.3)	69.34	210(3.1)	74.27	79(1.2)	77.82
**Radiation**											
Yes	33110	26791(100)	68.68	14168(52.9)	68.13	10984(41.0)	68.48	1252(4.7)	73.96	387(1.4)	77.67
**Chemotherapy**											
Yes	36637	29499(100)	67.09	15133(51.3)	66.17	12636(42.8)	67.18	1331(4.5)	73.55	399(1.4)	77.89

**Table 2:** Standardized Mortality Ratio (SMR) for each cause of death after Esophageal Cancer diagnosis

Cause of Death	Less than a year		1-5 years		5-10 years		More than 10 years		Total	
	Observed^1^	SMR (95% CI^2^)	Observed^1^	SMR (95% CI^2^)	Observed^1^	SMR (95% CI^2^)	Observed^1^	SMR (95% CI^2^)	Observed^1^	SMR (95% CI^2^)
All causes of death	32,923	26.40# (26.11-26.68)	16,731	10.91# (10.75-11.08)	2,134	2.80# (2.68-2.92)	715	1.98# (1.83-2.13)	52,503	13.45# (13.33-13.56)
All malignant cancer causes of death	29,696	97.64# (96.53-98.76)	14,558	38.35# (37.73-38.98)	1,170	6.51# (6.15-6.90)	293	3.77# (3.35-4.23)	45,717	48.58# (48.14-49.03)
Esophageal Cancer Deaths	26,873	2,849.79# (2,815.82-2,884.07)	12,980	1,083.01# (1,064.46-1,101.80)	800	142.82# (133.10-153.07)	156	66.87# (56.79-78.22)	40,809	1,390.47# (1,377.01-1,404.02)
**Non-cancer causes of death**										
*In situ*, benign or unknown behavior neoplasm	75	9.29# (7.31-11.65)	47	4.63# (3.4-6.15)	15	2.86# (1.60-4.72)	1	0.40 (0.01-2.21)	138	5.31# (4.46-6.27)
Tuberculosis	1	3.02 (0.08-16.82)	0	0.00 (0.00-9.90)	1	6.01 (0.15-33.49)	0	0.00 (0.00-54.28)	2	2.13 (0.26-7.70)
Syphilis	0	0.00 (0.00-181.54)	0	0.00 (0.00-153.88)	0	0.00 (0.00-313.98)	0	0.00 (0.00-721.58)	0	0.00 (0.00-60.32)
Septicemia	136	7.61# (6.38-9.00)	92	4.16# (3.35-5.10)	31	2.80# (1.90-3.97)	12	2.29# (1.18-4.01)	271	4.81# (4.26-5.42)
Other Infectious Diseases	79	7.50# (5.94-9.35)	38	2.94# (2.08-4.04)	12	1.98# (1.02-3.46)	1	0.39 (0.01-2.18)	130	4.06# (3.39-4.82)
Diabetes Mellitus	76	1.97# (1.55-2.47)	52	1.10 (0.82-1.44)	16	0.70 (0.40-1.13)	7	0.68 (0.27-1.40)	151	1.27# (1.07-1.49)
Alzheimer's	24	0.63# (0.40-0.94)	32	0.67# (0.46-0.94)	32	1.13 (0.77-1.60)	19	1.13 (0.68-1.77)	107	0.82# (0.67-0.99)
Diseases of Heart	1,081	3.12# (2.93-3.31)	755	1.82# (1.70-1.96)	349	1.73# (1.56-1.93)	154	1.61# (1.37-1.89)	2,339	2.21# (2.12-2.30)
Hypertension without Heart Disease	43	3.36# (2.43-4.52)	26	1.63# (1.07-2.39)	13	1.51 (0.80-2.57)	7	1.57 (0.63-3.23)	89	2.13# (1.71-2.62)
Cerebrovascular Diseases	180	2.53# (2.17-2.93)	106	1.27# (1.04-1.53)	58	1.42# (1.08-1.83)	22	1.09 (0.69-1.66)	366	1.70# (1.53-1.88)
Atherosclerosis	20	4.32# (2.64-6.67)	9	1.80 (0.82-3.41)	2	0.92 (0.11-3.31)	1	1.05 (0.03-5.85)	32	2.50# (1.71-3.54)
Aortic Aneurysm and Dissection	20	2.81# (1.72-4.34)	15	1.82# (1.02-3.00)	2	0.56 (0.07-2.03)	2	1.37 (0.17-4.94)	39	1.91# (1.36-2.61)
Other Diseases of Arteries, Arterioles, Capillaries	12	2.42# (1.25-4.24)	10	1.68 (0.81-3.09)	4	1.37 (0.37-3.52)	3	2.18 (0.45-6.38)	29	1.91# (1.28-2.74)
Pneumonia and Influenza	130	4.25# (3.55-5.05)	81	2.25# (1.79-2.80)	38	2.13# (1.51-2.93)	17	1.98# (1.15-3.17)	266	2.86# (2.53-3.23)
Chronic Obstructive Pulmonary Disease	273	3.54# (3.13-3.98)	243	2.48# (2.18-2.82)	107	2.16# (1.77-2.62)	55	2.38# (1.79-3.10)	678	2.74# (2.54-2.95)
Stomach and Duodenal Ulcers	6	3.40# (1.25-7.40)	5	2.44 (0.79-5.69)	2	2.13 (0.26-7.69)	1	2.35 (0.06-13.09)	14	2.70# (1.48-4.53)
Chronic Liver Disease and Cirrhosis	84	6.26# (5.00-7.76)	31	1.83# (1.25-2.60)	15	2.00# (1.12-3.30)	7	2.31 (0.93-4.75)	137	3.35# (2.82-3.96)
Nephritis, Nephrotic Syndrome and Nephrosis	70	2.80# (2.18-3.53)	38	1.23 (0.87-1.68)	18	1.13 (0.67-1.79)	10	1.32 (0.63-2.43)	136	1.71# (1.44-2.03)
Complications of Pregnancy, Childbirth, Puerperium	2	349.51# (42.33-1,262.53)	0	0.00 (0.00-562.86)	0	0.00 (0.00-1,731.74)	0	0.00 (0.00-6,411.75)	2	133.50# (16.17-482.23)
Congenital Anomalies	4	3.74# (1.02-9.57)	1	0.77 (0.02-4.28)	1	1.74 (0.04-9.71)	0	0.00 (0.00-15.29)	6	1.88 (0.69-4.10)
Certain Conditions Originating in Perinatal Period	0	0.00 (0.00-784.21)	1	171.64# (4.35-956.30)	0	0.00 (0.00-1,455.81)	0	0.00 (0.00-3,550.19)	1	70.91# (1.80-395.06)
Symptoms, Signs and Ill-Defined Conditions	81	6.36# (5.05-7.90)	47	3.00# (2.21-3.99)	9	1.09 (0.50-2.06)	8	2.04 (0.88-4.01)	145	3.57# (3.01-4.20)
Accidents and Adverse Effects	67	1.89# (1.47-2.40)	70	1.59# (1.24-2.01)	38	1.74# (1.23-2.39)	12	1.15 (0.59-2.00)	187	1.67# (1.44-1.93)
Suicide and Self-Inflicted Injury	89	9.16# (7.36-11.27)	33	2.70# (1.86-3.79)	8	1.46 (0.63-2.88)	4	1.79 (0.49-4.59)	134	4.52# (3.79-5.36)
Homicide and Legal Intervention	0	0.00 (0.00-2.39)	6	3.52# (1.29-7.65)	0	0.00 (0.00-5.70)	0	0.00 (0.00-15.83)	6	1.45 (0.53-3.16)
Other Cause of Death	674	3.88# (3.59-4.18)	435	1.97# (1.79-2.16)	193	1.60# (1.38-1.84)	79	1.26 (1.00-1.57)	1381	2.39# (2.26-2.52)

**1** number of cancer patients who died due to each cause of death. **2** 95% Confidence interval. **#** P value less than .05.
