# Supplementary material for: What Causes Death in Esophageal Cancer Patients Other Than the Cancer Itself: A Large Population-Based Analysis
Source: J Cancer. 2022 Oct 17;13(13):3485–94. doi: 10.7150/jca.78004 (PMC9608205; doi:10.7150/jca.78004)
Supplement: Supplementary file 1 — Supplementary tables. [file jcav13p3485s1.pdf]

Supplementary table 1: Standardized mortality ratio (SMR) for each cause of death following esophageal cancer diagnosis aged younger than 49 years.

Cause of Death	Less than a year		1-5 years		5-10 years		More than 10 years		Total	
	Observed[1]	SMR (95% CI[2])	Observed[1]	SMR (95% CI[2])	Observed[1]	SMR (95% CI[2])	Observed[1]	SMR (95% CI[2])	Observed[1]	SMR (95% CI[2])
All Causes of Death	1,902	200.77# (191.84-210.00)	873	104.41# (97.60-111.58)	34	17.64# (12.21-24.65)	5	10.06# (3.27-23.48)	2,814	138.90# (133.81-144.13)
All Malignant Cancer Causes of Death	1,807	1,055.87# (1,007.75-1,105.71)	824	549.82# (512.91-588.67)	26	78.41# (51.22-114.88)	4	46.17# (12.58-118.21)	2,661	733.40# (705.80-761.81)
Esophageal Cancer Deaths	1,614	24,276.35# (23,106.31-25,490.29)	729	12,378.04# (11,495.69-13,310.15)	20	1,496.17# (913.90-2,310.72)	3	920.12# (189.75-2,688.98)	2,366	16,661.16# (15,996.51-17,346.33)
Non-cancer Causes of Death										
In situ, benign or unknown behavior neoplasm	1	31.39 (0.79-174.91)	3	107.00# (22.07-312.70)	0	0.00 (0.00-583.96)	0	0.00 (0.00-2365.55)	4	59.02# (16.08-151.13)
Tuberculosis	0	0.00 (0.00-869.69)	0	0.00 (0.00-1,095.16)	0	0.00 (0.00-5,658.25)	0	0.00 (0.00-24,248.05)	0	0.00 (0.00-438.42)
Syphilis	0	0.00 (0.00-17,197.57)	0	0.00 (0.00-23,294.4	0	0.00 (0.00-119,560	0	0.00 (0.00-432,131	0	0.00 (0.00-8,948.

				0)		.57)		.12)		18)
Septicemia	4	39.56# (10.78-101.29)	1	11.40 (0.29-63.50)	0	0.00 (0.00-183.00)	0	0.00 (0.00-674.72)	5	23.31# (7.57-54.40)
Other Infectious Diseases	6	13.58# (4.98-29.55)	1	2.96 (0.07-16.48)	0	0.00 (0.00-62.26)	0	0.00 (0.00-323.40)	7	8.23# (3.31-16.96)
Diabetes Mellitus	0	0.00 (0.00-13.58)	0	0.00 (0.00-15.38)	0	0.00 (0.00-64.99)	0	0.00 (0.00-237.10)	0	0.00 (0.00-6.32)
Alzheimer's	0	0.00 (0.00-1,834.07)	1	530.60# (13.43-2,956.30)	0	0.00 (0.00-8,102.05)	0	0.00 (0.00-31,364.67)	1	223.77# (5.67-1,246.76)
Diseases of Heart	24	12.89# (8.26-19.18)	16	9.95# (5.69-16.16)	1	2.79 (0.07-15.52)	1	11.10 (0.28-61.84)	42	10.72# (7.72-14.49)
Hypertension without Heart Disease	1	16.79 (0.42-93.52)	0	0.00 (0.00-73.53)	0	0.00 (0.00-318.05)	0	0.00 (0.00-1,129.20)	1	8.02 (0.20-44.71)
Cerebrovascular Diseases	4	15.15# (4.13-38.79)	2	9.02# (1.09-32.58)	1	20.89 (0.53-116.38)	0	0.00 (0.00-291.02)	7	12.81# (5.15-26.40)
Atherosclerosis	0	0.00 (0.00-625.31)	0	0.00 (0.00-754.07)	0	0.00 (0.00-3,799.88)	0	0.00 (0.00-16,495.59)	0	0.00 (0.00-307.77)
Aortic Aneurysm and Dissection	0	0.00 (0.00-83.83)	0	0.00 (0.00-97.46)	0	0.00 (0.00-424.55)	0	0.00 (0.00-1,672.57)	0	0.00 (0.00-39.77)

Other Diseases of Arteries, Arterioles, Capillaries	0	0.00 (0.00-244.34)	0	0.00 (0.00-283.43)	0	0.00 (0.00-1,251.08)	0	0.00 (0.00-4,370.12)	0	0.00 (0.00-115.62)
Pneumonia and Influenza	5	48.10# (15.62-112.24)	3	32.83# (6.77-95.95)	0	0.00 (0.00-173.76)	0	0.00 (0.00-666.69)	8	36.02# (15.55-70.97)
Chronic Obstructive Pulmonary Disease	4	32.77# (8.93-83.90)	0	0.00 (0.00-34.28)	0	0.00 (0.00-151.46)	0	0.00 (0.00-575.53)	4	15.36# (4.18-39.32)
Stomach and Duodenal Ulcers	0	0.00 (0.00-279.64)	0	0.00 (0.00-328.61)	0	0.00 (0.00-1,524.35)	0	0.00 (0.00-5,990.67)	0	0.00 (0.00-134.37)
Chronic Liver Disease and Cirrhosis	2	4.58 (0.55-16.53)	1	2.52 (0.06-14.02)	1	10.55 (0.27-58.80)	0	0.00 (0.00-143.92)	4	4.19# (1.14-10.73)
Nephritis, Nephrotic Syndrome and Nephrosis	2	20.96# (2.54-75.72)	0	0.00 (0.00-46.15)	0	0.00 (0.00-209.12)	0	0.00 (0.00-784.80)	2	10.12# (1.23-36.55)
Complications of Pregnancy, Childbirth, Puerperium	1	338.99# (8.58-1,888.74)	0	0.00 (0.00-1,144.15)	0	0.00 (0.00-3,667.62)	0	0.00 (0.00-11,879.71)	1	133.50# (3.38-743.84)

Congenital Anomalies	0	0.00 (0.00-89.07)	0	0.00 (0.00-98.09)	0	0.00 (0.00-412.91)	0	0.00 (0.00-1,578.1)	0	0.00 (0.00-40.85)
Certain Conditions Originating in Perinatal Period	0	0.00 (0.00-10,089.17)	0	0.00 (0.00-11,042.2 7)	0	0.00 (0.00-47,470. 08)	0	0.00 (0.00-222,709 .26)	0	0.00 (0.00-4,646. 12)
Symptoms, Signs and Ill-Defined Conditions	5	29.23# (9.49-68.21)	2	13.85# (1.68-50.02)	0	0.00 (0.00-122.92)	0	0.00 (0.00-521.37)	7	19.85# (7.98-40.90)
Accidents and Adverse Effects	5	3.09# (1.00-7.21)	5	3.32# (1.08-7.76)	1	2.65 (0.07-14.76)	0	0.00 (0.00-37.11)	11	3.06# (1.53-5.47)
Suicide and Self-Inflicted Injury	3	4.38 (0.90-12.80)	2	3.07 (0.37-11.09)	0	0.00 (0.00-22.15)	0	0.00 (0.00-86.60)	5	3.23# (1.05-7.55)
Homicide and Legal Intervention	0	0.00 (0.00-15.78)	0	0.00 (0.00-19.06)	0	0.00 (0.00-86.35)	0	0.00 (0.00-359.32)	0	0.00 (0.00-7.68)
Other Cause of Death	28	24.67# (16.39-35.65)	12	11.94# (6.17-20.86)	4	17.07# (4.65-43.71)	0	0.00 (0.00-59.62)	44	18.06# (13.12-24.25)

1 number of cancer patients who died due to each cause of death.

2 95% Confidence interval.

# P value less than .05.

Supplementary table 2: Standardized mortality ratio (SMR) for each cause of death following esophageal cancer diagnosis aged 50-64 years.

Cause of Death	Less than a year		1-5 years		5-10 years		More than 10 years		Total	
	Observed[1]	SMR (95% CI[2])	Observed[1]	SMR (95% CI[2])	Observed[1]	SMR (95% CI[2])	Observed[1]	SMR (95% CI[2])	Observed[1]	SMR (95% CI[2])
All Causes of Death	10,149	68.64# (67.32-69.99)	5,434	31.95# (31.11-32.81)	348	5.76# (5.17-6.39)	53	2.94# (2.20-3.85)	15,984	40.32# (39.70-40.95)
All Malignant Cancer Causes of Death	9,393	201.70# (197.64-205.82)	4,998	93.11# (90.55-95.73)	228	12.04# (10.53-13.71)	25	4.55# (2.95-6.72)	14,644	117.46# (115.57-119.38)
Esophageal Cancer Deaths	8,443	4,130.76# (4,043.12-4,219.83)	4,482	1,895.07# (1,839.99-1,951.38)	159	189.22# (160.95-221.03)	13	52.67# (28.04-90.06)	13,097	2,382.95# (2,342.31-2,424.11)
Non-cancer Causes of Death										
In situ, benign or unknown behavior neoplasm	18	28.03# (16.61-44.30)	10	13.41# (8.43-24.66)	2	7.57 (0.92-27.33)	1	12.96 (0.33-72.18)	31	17.92# (12.18-25.44)
Tuberculosis	0	0.00 (0.00-66.28)	0	0.00 (0.00-63.73)	0	0.00 (0.00-199.54)	0	0.00 (0.00-728.26)	0	0.00 (0.00-26.91)
Syphilis	0	0.00	0	0.00	0	0.00	0	0.00	0	0.00

		(0.00-1,078.99)		(0.00-1,022.06)		(0.00-2,944.7 2)		(0.00-9,30)		(0.00-425.13)
Septicemia	41	20.15# (14.46-27.34)	25	10.62# (6.87-15.68)	5	5.82# (1.89-13.57)	2	7.63 (0.92-27.56)	73	13.25# (10.39-16.66)
Other Infectious Diseases	39	12.20# (8.67-16.67)	20	5.66# (3.45-8.74)	3	2.45 (0.51-7.17)	0	0.00 (0.00-10.78)	62	7.47# (5.73-9.58)
Diabetes Mellitus	16	2.80# (1.60-4.55)	3	0.46 (0.09-1.34)	2	0.86 (0.10-3.10)	0	0.00 (0.00-5.23)	21	1.37 (0.85-2.10)
Alzheimer’s	1	3.53 (0.09-19.66)	0	0.00 (0.00-10.43)	0	0.00 (0.00-26.65)	0	0.00 (0.00-79.50)	1	1.22 (0.03-6.78)
Diseases of Heart	206	5.58# (4.85-6.40)	109	2.62# (2.15-3.16)	42	2.92# (2.11-3.95)	11	2.59# (1.30-4.64)	368	3.79# (3.41-4.20)
Hypertension without Heart Disease	11	8.63# (4.31-15.45)	5	3.41# (1.11-7.95)	1	1.81 (0.05-10.10)	1	5.60 (0.14-31.20)	18	5.18# (3.07-8.19)
Cerebrovascular Diseases	35	6.86# (4.78-9.54)	19	3.35# (2.02-5.23)	3	1.53 (0.31-4.46)	1	1.72 (0.04-9.59)	58	4.35# (3.31-5.63)
Atherosclerosis	4	20.24# (5.52-51.83)	1	4.62 (0.12-25.72)	0	0.00 (0.00-52.07)	0	0.00 (0.00-186.15)	5	9.90# (3.22-23.11)
Aortic Aneurysm and Dissection	3	4.03 (0.83-11.79)	1	1.21 (0.03-6.77)	0	0.00 (0.00-13.50)	0	0.00 (0.00-47.53)	4	2.09 (0.57-5.34)
Other Diseases of Arteries, Arterioles,	5	12.06# (3.92-28.15)	1	2.10 (0.05-11.69)	1	5.83 (0.15-32.47)	0	0.00 (0.00-69.22)	7	6.27# (2.52-12.92)

Capillaries										
Pneumonia and Influenza	27	14.77# (9.73-21.49)	18	8.49# (5.03-13.42)	3	3.89 (0.80-11.38)	1	4.22 (0.11-23.51)	49	9.89# (7.31-13.07)
Chronic Obstructive Pulmonary Disease	47	7.93# (5.83-10.55)	32	4.51# (3.08-6.37)	10	3.80# (1.82-6.99)	3	3.67 (0.76-10.72)	92	5.59# (4.50-6.85)
Stomach and Duodenal Ulcers	1	4.53 (0.11-25.23)	2	8.02 (0.97-28.98)	1	11.52 (0.29-64.19)	0	0.00 (0.00-139.60)	4	6.86# (1.87-17.56)
Chronic Liver Disease and Cirrhosis	39	7.54# (5.36-10.30)	20	3.27# (2.00-5.05)	3	1.33 (0.27-3.88)	2	2.87 (0.35-10.36)	64	4.49# (3.46-5.73)
Nephritis, Nephrotic Syndrome and Nephrosis	19	8.92# (5.37-13.93)	8	3.32# (1.43-6.55)	2	2.32 (0.28-8.38)	0	0.00 (0.00-14.29)	29	5.13# (3.43-7.36)
Complications of Pregnancy, Childbirth, Puerperium	1	371.69# (9.41-2,070.92)	0	0.00 (0.00-1,149.53)	0	0.00 (0.00-3,483.91)	0	0.00 (0.00-14,844.09)	1	138.76# (3.51-773.11)
Congenital Anomalies	3	8.30# (1.71-24.25)	0	0.00 (0.00-8.60)	0	0.00 (0.00-23.50)	0	0.00 (0.00-75.74)	3	3.01 (0.62-8.80)
Certain Conditions	0	0.00	0	0.00	0	0.00	0	0.00	0	0.00

Originating in Perinatal Period		(0.00-2,347.01)		(0.00-1,931.93)		(0.00-5,193.54)		(0.00-15,872.51)		(0.00-833.86)
Symptoms, Signs and Ill-Defined Conditions	20	14.73# (9.00-22.75)	14	9.12# (4.98-15.29)	1	1.87 (0.05-10.42)	1	6.34 (0.16-35.32)	36	10.04# (7.03-13.90)
Accidents and Adverse Effects	18	2.24# (1.33-3.54)	21	2.23# (1.38-3.41)	10	2.90# (1.39-5.33)	0	0.00 (0.00-3.43)	49	2.23# (1.65-2.95)
Suicide and Self-Inflicted Injury	15	4.33# (2.42-7.14)	11	2.65# (1.32-4.74)	3	1.97 (0.41-5.75)	0	0.00 (0.00-8.14)	29	3.02# (2.02-4.34)
Homicide and Legal Intervention	0	0.00 (0.00-5.21)	3	3.97 (0.82-11.60)	0	0.00 (0.00-14.71)	0	0.00 (0.00-50.26)	3	1.68 (0.35-4.90)
Other Cause of Death	187	12.05# (10.38-13.91)	113	6.18# (5.09-7.43)	28	4.14# (2.75-5.99)	5	2.39 (0.78-5.57)	333	7.81# (6.99-8.69)

1 number of cancer patients who died due to each cause of death.

2 95% Confidence interval.

# P value less than .05.

Supplementary table 3: Standardized mortality ratio (SMR) for each cause of death following esophageal cancer diagnosis aged older than 64 years.

Cause of Death	Less than a year		1-5 years		5-10 years		More than 10 years		Total	

	Observed[1]	SMR (95% CI[2])	Observed[1]	SMR (95% CI[2])	Observed[1]	SMR (95% CI[2])	Observed[1]	SMR (95% CI[2])	Observed[1]	SMR (95% CI[2])
All Causes of Death	20,872	19.15# (18.89-19.41)	10,424	7.69# (7.55-7.84)	1,752	2.50# (2.39-2.62)	657	1.91# (1.77-2.07)	33,705	9.66# (9.56-9.77)
All Malignant Cancer Causes of Death	18,496	72.29# (71.25-73.34)	8,736	26.93# (26.36-27.50)	916	5.71# (5.35-6.09)	264	3.66# (3.23-4.13)	28,412	34.96# (34.55-35.37)
Esophageal Cancer Deaths	16,816	2,297.46# (2,262.86-2,332.45)	7,769	812.56# (794.59-830.83)	621	130.80# (120.72-141.51)	140	67.22# (56.54-79.32)	25,346	1,068.95# (1,055.83-1,082.20)
Non-cancer Causes of Death										
In situ, benign or unknown behavior neoplasm	56	7.57# (5.72-9.83)	34	3.62# (2.51-5.06)	13	2.61 (1.39-4.47)	0	0.00 (0.00-1.51)	103	4.26# (3.47-5.16)
Tuberculosis	1	3.69 (0.09-20.53)	0	0.00 (0.00-11.84)	1	6.79 (0.17-37.84)	0	0.00 (0.00-58.79)	2	2.52 (0.31-9.11)
Syphilis	0	0.00 (0.00-221.07)	0	0.00 (0.00-182.57)	0	0.00 (0.00-352.49)	0	0.00 (0.00-783.64)	0	0.00 (0.00-70.85)
Septicemia	91	5.78# (4.65-7.10)	34	3.62# (2.51-5.06)	13	2.61# (1.39-4.47)	0	0.00 (0.00-1.51)	193	3.81# (3.30-4.39)

Other Infectious Diseases	34	4.93# (3.42-6.90)	0	0.00 (0.00-11.84)	1	6.79 (0.17-37.84)	0	0.00 (0.00-58.79)	61	2.66# (2.04-3.42)
Diabetes Mellitus	60	1.84# (1.41-2.37)	0	0.00 (0.00-182.57)	0	0.00 (0.00-352.49)	0	0.00 (0.00-783.64)	130	1.26# (1.05-1.49)
Alzheimer's	23	0.61# (0.39-0.91)	66	3.35# (2.59-4.26)	26	2.55# (1.67-3.74)	10	2.01 (0.97-3.70)	105	0.81# (0.66-0.98)
Diseases of Heart	851	2.76# (2.58-2.96)	17	1.88# (1.10-3.01)	9	1.88 (0.86-3.58)	1	0.45 (0.01-2.52)	1,929	2.02# (1.93-2.11)
Hypertension without Heart Disease	31	2.70# (1.84-3.83)	49	1.20 (0.89-1.59)	14	0.68 (0.37-1.15)	7	0.73 (0.29-1.51)	70	1.83# (1.43-2.31)
Cerebrovascular Diseases	141	2.14# (1.80-2.53)	31	0.65# (0.44-0.92)	32	1.14 (0.78-1.60)	19	1.14 (0.68-1.77)	301	1.49# (1.33-1.67)
Atherosclerosis	16	3.61# (2.06-5.86)	630	1.70# (1.57-1.84)	306	1.64# (1.46-1.84)	142	1.56# (1.31-1.83)	27	2.20# (1.45-3.20)
Aortic Aneurysm and Dissection	17	2.69# (1.57-4.30)	21	1.46 (0.90-2.23)	12	1.49 (0.77-2.60)	6	1.40 (0.51-3.05)	35	1.90# (1.33-2.65)
Other Diseases of Arteries, Arterioles,	7	1.55 (0.62-3.19)	85	1.09 (0.87-1.35)	54	1.39# (1.04-1.81)	21	1.08 (0.67-1.65)	22	1.57 (0.98-2.37)

Capillaries										
Pneumonia and Influenza	98	3.42#<br>(2.78-4.17)	8	1.67<br>(0.72-3.29)	2	0.95<br>(0.11-3.43)	1	1.07<br>(0.03-5.97)	209	2.38#<br>(2.07-2.73)
Chronic Obstructive Pulmonary Disease	222	3.12#<br>(2.72-3.56)	14	1.89#<br>(1.04-3.18)	2	0.61<br>(0.07-2.20)	2	1.45<br>(0.18-5.22)	582	2.52#<br>(2.32-2.73)
Stomach and Duodenal Ulcers	5	3.27#<br>(1.06-7.63)	9	1.65<br>(0.75-3.13)	3	1.10<br>(0.23-3.20)	3	2.27<br>(0.47-6.64)	10	2.19#<br>(1.05-4.02)
Chronic Liver Disease and Cirrhosis	43	5.51#<br>(3.99-7.43)	60	1.78#<br>(1.36-2.29)	35	2.05#<br>(1.43-2.86)	16	1.92#<br>(1.10-3.11)	69	2.69#<br>(2.09-3.41)
Nephritis, Nephrotic Syndrome and Nephrosis	49	2.15#<br>(1.59-2.84)	211	2.33#<br>(2.02-2.66)	97	2.07#<br>(1.68-2.53)	52	2.33#<br>(1.74-3.06)	105	1.43#<br>(1.17-1.73)
Complications of Pregnancy, Childbirth, Puerperium	0	0.00<br>(0.00-44,967.46)	3	1.68<br>(0.35-4.90)	1	1.18<br>(0.03-6.55)	1	2.51<br>(0.06-13.98)	0	0.00<br>(0.00-12,963.34)
Congenital Anomalies	1	1.50<br>(0.04-8.36)	10	0.96<br>(0.46-1.77)	11	2.14#<br>(1.07-3.83)	5	2.16<br>(0.70-5.05)	3	1.43<br>(0.29-4.17)

Certain Conditions Originating in Perinatal Period	0	0.00 (0.00-1,333.36)	30	1.05 (0.71-1.50)	16	1.07 (0.61-1.73)	10	1.37 (0.66-2.52)	1	112.55# (2.85-627.06)
Symptoms, Signs and Ill-Defined Conditions	56	5.00# (3.77-6.49)	0	0.00 (0.00-30,563.59)	0	0.00 (0.00-56,293.58)	0	0.00 (0.00-226,263.15)	102	2.78# (2.27-3.38)
Accidents and Adverse Effects	44	1.71# (1.24-2.29)	1	1.20 (0.03-6.66)	1	2.45 (0.06-13.66)	0	0.00 (0.00-19.40)	127	1.47# (1.23-1.75)
Suicide and Self-Inflicted Injury	71	12.76# (9.96-16.09)	1	279.12# (7.07-1,555.14)	0	0.00 (0.00-2,112.87)	0	0.00 (0.00-4,668.90)	100	5.41# (4.40-6.58)
Homicide and Legal Intervention	0	0.00 (0.00-6.11)	31	2.22# (1.51-3.15)	8	1.04 (0.45-2.04)	7	1.86 (0.75-3.83)	3	1.61 (0.33-4.71)
Other Cause of Death	459	2.92# (2.66-3.20)	310	1.54# (1.37-1.72)	161	1.41# (1.20-1.65)	74	1.22 (0.96-1.53)	1004	1.88# (1.77-2.00)

1 number of cancer patients who died due to each cause of death.

2 95% Confidence interval.

# P value less than .05.

Supplementary table 4: Standardized mortality ratio (SMR) for each cause of death following esophagus cancer diagnosis in male.

Cause of Death	Less than a year		1-5 years		5-10 years		More than 10 years		Total	
	Observed[1]	SMR (95% CI[2])	Observed[1]	SMR (95% CI[2])	Observed[1]	SMR (95% CI[2])	Observed[1]	SMR (95% CI[2])	Observed[1]	SMR (95% CI[2])
All Causes of Death	25,145	26.21# (25.89-26.54)	13,181	11.08# (10.89-11.27)	1,643	2.80# (2.66-2.94)	519	1.89# (1.73-2.06)	40,488	13.45# (13.32-13.58)
All Malignant Cancer Causes of Death	22,692	91.92# (90.73-93.12)	11,508	37.36# (36.68-38.05)	914	6.27# (5.87-6.69)	218	3.48# (3.03-3.97)	35,332	46.29# (45.81-46.77)
Esophageal Cancer Deaths	20,536	2,340.23# (2,308.33-2,372.46)	10,258	917.96# (900.28-935.90)	626	119.86# (110.65-129.63)	118	54.47# (45.09-65.23)	31,538	1,153.58# (1,140.89-1,166.39)
Non-cancer Causes of Death										
In situ, benign or unknown behavior neoplasm	54	8.42# (6.33-10.99)	37	4.54# (3.19-6.25)	12	2.84# (1.47-4.96)	1	0.50 (0.01-2.76)	104	5.00# (4.08-6.06)
Tuberculosis	1	3.61 (0.09-20.11)	0	0.00 (0.00-11.85)	0	0.00 (0.00-26.59)	0	0.00 (0.00-65.29)	1	1.28 (0.03-7.11)
Syphilis	0	0.00	0	0.00	0	0.00	0	0.00	0	0.00

		(0.00-218.66)		(0.00-182.11)		(0.00-373.18)		(0.00-893.40)		(0.00-72.13)
Septicemia	105	7.82# (6.39-9.46)	69	4.11# (3.20-5.20)	21	2.50# (1.55-3.82)	9	2.28# (1.04-4.33)	204	4.79# (4.16-5.50)
Other Infectious Diseases	65	7.63# (5.89-9.72)	30	2.89# (1.95-4.13)	8	1.68 (0.72-3.30)	1	0.50 (0.01-2.81)	104	4.06# (3.31-4.91)
Diabetes Mellitus	56	1.86# (1.41-2.42)	46	1.23 (0.90-1.64)	14	0.77 (0.42-1.30)	4	0.49 (0.13-1.26)	120	1.28# (1.06-1.53)
Alzheimer’s	11	0.48# (0.24-0.86)	21	0.71 (0.44-1.08)	20	1.14 (0.70-1.76)	16	1.57 (0.90-2.55)	68	0.85 (0.66-1.07)
Diseases of Heart	848	3.15# (2.95-3.37)	580	1.78# (1.64-1.93)	274	1.74# (1.54-1.96)	107	1.45# (1.19-1.75)	1,809	2.19# (2.09-2.29)
Hypertension without Heart Disease	25	2.82# (1.82-4.16)	17	1.51 (0.88-2.42)	9	1.49 (0.68-2.83)	5	1.62 (0.53-3.79)	56	1.92# (1.45-2.49)
Cerebrovascular Diseases	136	2.74# (2.30-3.24)	79	1.33# (1.05-1.66)	31	1.07 (0.73-1.53)	15	1.08 (0.61-1.78)	261	1.72# (1.52-1.94)
Atherosclerosis	15	4.72# (2.64-7.78)	7	1.98 (0.79-4.07)	1	0.65 (0.02-3.62)	0	0.00 (0.00-5.54)	23	2.58# (1.63-3.87)
Aortic Aneurysm and Dissection	18	3.05# (1.81-4.82)	15	2.19# (1.22-3.61)	2	0.68 (0.08-2.47)	2	1.70 (0.21-6.14)	37	2.19# (1.55-3.02)

Other Diseases of Arteries, Arterioles, Capillaries	9	2.46# (1.12-4.66)	10	2.23# (1.07-4.11)	4	1.83 (0.50-4.70)	1	0.99 (0.02-5.50)	24	2.12# (1.36-3.15)
Pneumonia and Influenza	94	4.12# (3.33-5.04)	63	2.31# (1.77-2.95)	29	2.14# (1.43-3.08)	13	2.01# (1.07-3.44)	199	2.84# (2.46-3.26)
Chronic Obstructive Pulmonary Disease	184	3.07# (2.65-3.55)	178	2.34# (2.01-2.71)	82	2.14# (1.70-2.66)	44	2.49# (1.81-3.35)	488	2.54# (2.32-2.78)
Stomach and Duodenal Ulcers	5	3.71# (1.20-8.65)	5	3.15# (1.02-7.36)	1	1.38 (0.03-7.69)	0	0.00 (0.00-11.42)	11	2.76# (1.38-4.94)
Chronic Liver Disease and Cirrhosis	73	6.24# (4.89-7.85)	27	1.84# (1.21-2.68)	13	2.01# (1.07-3.43)	6	2.31 (0.85-5.03)	119	3.36# (2.78-4.02)
Nephritis, Nephrotic Syndrome and Nephrosis	53	2.74# (2.05-3.58)	29	1.20 (0.80-1.72)	15	1.21 (0.68-2.00)	8	1.36 (0.59-2.68)	105	1.70# (1.39-2.06)
Complications of Pregnancy, Childbirth, Puerperium	0	0.00 (0.00-0.00)	0	0.00 (0.00-0.00)	0	0.00 (0.00-0.00)	0	0.00 (0.00-0.00)	0	0.00 (0.00-0.00)

Congenital Anomalies	4	4.75# (1.29-12.15)	0	0.00 (0.00-3.60)	1	2.23 (0.06-12.41)	0	0.00 (0.00-19.75)	5	2.00 (0.65-4.66)
Certain Conditions Originating in Perinatal Period	0	0.00 (0.00-882.86)	1	193.89# (4.91-1,080.30)	0	0.00 (0.00-1,656.39)	0	0.00 (0.00-4152.87)	1	80.31# (2.03-447.48)
Symptoms, Signs and Ill-Defined Conditions	60	6.82# (5.20-8.77)	37	3.36# (2.36-4.63)	7	1.22 (0.49-2.52)	6	2.24 (0.82-4.88)	110	3.90# (3.20-4.70)
Accidents and Adverse Effects	45	1.55# (1.13-2.07)	62	1.71# (1.31-2.19)	29	1.64# (1.10-2.35)	11	1.32 (0.66-2.37)	147	1.61# (1.36-1.89)
Suicide and Self-Inflicted Injury	87	9.42# (7.55-11.62)	32	2.76# (1.89-3.90)	8	1.54 (0.67-3.04)	4	1.89 (0.51-4.83)	131	4.66# (3.90-5.53)
Homicide and Legal Intervention	0	0.00 (0.00-2.67)	5	3.32# (1.08-7.74)	0	0.00 (0.00-6.55)	0	0.00 (0.00-18.46)	5	1.37 (0.44-3.20)
Other Cause of Death	505	4.00# (3.65-4.36)	323	1.98# (1.77-2.21)	148	1.67# (1.41-1.96)	48	1.06 (0.78-1.41)	1024	2.42# (2.27-2.57)

1 number of cancer patients who died due to each cause of death.

2 95% Confidence interval.

# P value less than .05.

Supplementary table 5: Standardized mortality ratio (SMR) for each cause of death following esophageal cancer diagnosis in female.

Cause of Death	Less than a year		1-5 years		5-10 years		More than 10 years		Total	
	Observed[1]	SMR (95% CI[2])	Observed[1]	SMR (95% CI[2])	Observed[1]	SMR (95% CI[2])	Observed[1]	SMR (95% CI[2])	Observed[1]	SMR (95% CI[2])
All Causes of Death	7,778	27.02# (26.42-27.63)	3,550	10.35# (10.01-10.69)	491	2.81# (2.57-3.07)	196	2.24# (1.94-2.58)	12,015	13.45# (13.21-13.70)
All Malignant Cancer Causes of Death	7,004	122.32# (119.47-125.22)	3,050	42.61# (41.11-44.15)	256	7.56# (6.66-8.54)	75	4.98# (3.92-6.24)	10,385	58.41# (57.3-59.55)
Esophageal Cancer Deaths	6,337	9,680.38# (9,443.49-9,921.71)	2,722	3,359.21# (3,234.19-3,487.82)	174	459.83# (394.04-533.46)	38	228.02# (161.36-312.98)	9,271	4,612.47# (4,519.05-4,707.33)
Non-cancer Causes of Death										
In situ, benign or unknown behavior neoplasm	21	12.68# (7.85-19.38)	10	4.99# (2.39-9.18)	3	2.94# (0.61-8.60)	0	0.00 (0.00-7.29)	34	6.56# (4.54-9.16)
Tuberculosis	0	0.00 (0.00-68.07)	0	0.00 (0.00-60.03)	1	36.15 (0.92-201.42)	0	0.00 (0.00-321.96)	1	6.46 (0.16-36.00)

Syphilis	0	0.00 (0.00-1,069.34)	0	0.00 (0.00-992.56)	0	0.00 (0.00-1,979.13)	0	0.00 (0.00-3,751.89)	0	0.00 (0.00-368.40)
Septicemia	31	6.97# (4.74-9.89)	23	4.30# (2.73-6.46)	10	3.73# (1.79-6.86)	3	2.34 (0.48-6.83)	67	4.87# (3.77-6.19)
Other Infectious Diseases	14	6.96# (3.81-11.68)	8	3.15# (1.36-6.21)	4	3.11 (0.85-7.96)	0	0.00 (0.00-6.37)	26	4.05# (2.65-5.94)
Diabetes Mellitus	20	2.37# (1.45-3.66)	6	0.60 (0.22-1.30)	2	0.42 (0.05-1.51)	3	1.38 (0.29-4.04)	31	1.22 (0.83-1.73)
Alzheimer's	13	0.86 (0.46-1.47)	11	0.60 (0.30-1.07)	12	1.12 (0.58-1.95)	3	0.46 (0.09-1.34)	39	0.77 (0.55-1.05)
Diseases of Heart	233	2.99# (2.62-3.40)	175	1.98# (1.70-2.30)	75	1.72# (1.35-2.15)	47	2.16# (1.58-2.87)	530	2.29# (2.10-2.49)
Hypertension without Heart Disease	18	4.58# (2.71-7.23)	9	1.92 (0.88-3.64)	4	1.54 (0.42-3.94)	2	1.44 (0.17-5.20)	33	2.61# (1.80-3.67)
Cerebrovascular Diseases	44	2.04# (1.48-2.74)	27	1.11 (0.73-1.61)	27	2.23# (1.47-3.24)	7	1.12 (0.45-2.32)	105	1.63# (1.34-1.98)
Atherosclerosis	5	3.44# (1.12-8.02)	2	1.36 (0.17-4.92)	1	1.56 (0.04-8.67)	1	3.49 (0.09-19.42)	9	2.34# (1.07-4.44)

Aortic Aneurysm and Dissection	2	1.65 (0.20-5.98)	0	0.00 (0.00-2.65)	0	0.00 (0.00-5.78)	0	0.00 (0.00-12.85)	2	0.57 (0.07-2.05)
Other Diseases of Arteries, Arterioles, Capillaries	3	2.34 (0.48-6.83)	0	0.00 (0.00-2.51)	0	0.00 (0.00-5.04)	2	5.53 (0.67-19.96)	5	1.30 (0.42-3.03)
Pneumonia and Influenza	36	4.64# (3.25-6.43)	18	2.08# (1.23-3.28)	9	2.10 (0.96-3.98)	4	1.88 (0.51-4.82)	67	2.93# (2.27-3.72)
Chronic Obstructive Pulmonary Disease	89	5.12# (4.12-6.31)	65	2.97# (2.29-3.78)	25	2.25# (1.46-3.32)	11	2.01# (1.00-3.59)	190	3.40# (2.94-3.92)
Stomach and Duodenal Ulcers	1	2.41 (0.06-13.43)	0	0.00 (0.00-7.94)	1	4.65 (0.12-25.88)	1	9.76 (0.25-54.36)	3	2.50 (0.52-7.32)
Chronic Liver Disease and Cirrhosis	11	6.42# (3.21-11.49)	4	1.79 (0.49-4.57)	2	1.95 (0.24-7.05)	1	2.28 (0.06-12.68)	18	3.32# (1.97-5.25)
Nephritis, Nephrotic Syndrome and Nephrosis	17	3.00# (1.74-4.80)	9	1.32 (0.60-2.51)	3	0.86 (0.18-2.50)	2	1.18 (0.14-4.27)	31	1.75# (1.19-2.49)
Complications of Pregnancy,	2	349.51# (42.33-1,262.53)	0	0.00 (0.00-562.86)	0	0.00 (0.00-1,731.7	0	0.00 (0.00-6,411.7	2	133.50# (16.17-482.2

Childbirth, Puerperium						4)		5)		3)
Congenital Anomalies	0	0.00 (0.00-16.25)	1	3.60 (0.09-20.06)	0	0.00 (0.00-29.58)	0	0.00 (0.00-67.75)	1	1.46 (0.04-8.15)
Certain Conditions Originating in Perinatal Period	0	0.00 (0.00-7,018.10)	0	0.00 (0.00-5,516.09)	0	0.00 (0.00-12,022.10)	0	0.00 (0.00-24,462.90)	0	0.00 (0.00-2,232.97)
Symptoms, Signs and Ill-Defined Conditions	21	5.34# (3.30-8.16)	10	2.16# (1.04-3.97)	2	0.78 (0.09-2.82)	2	1.59 (0.19-5.76)	35	2.83# (1.97-3.93)
Accidents and Adverse Effects	22	3.49# (2.19-5.29)	8	1.03 (0.44-2.03)	9	2.20# (1.01-4.18)	1	0.46 (0.01-2.58)	40	1.97# (1.41-2.68)
Suicide and Self-Inflicted Injury	2	4.13 (0.50-14.94)	1	1.56 (0.04-8.68)	0	0.00 (0.00-13.17)	0	0.00 (0.00-33.05)	3	1.98 (0.41-5.78)
Homicide and Legal Intervention	0	0.00 (0.00-22.31)	1	5.02 (0.13-27.95)	0	0.00 (0.00-43.94)	0	0.00 (0.00-111.49)	1	2.08 (0.05-11.57)
Other Cause of Death	169	3.57# (3.05-4.15)	112	1.93# (1.59-2.32)	45	1.39# (1.02-1.86)	31	1.77 (1.20-2.51)	357	2.30# (2.07-2.55)

1 number of cancer patients who died due to each cause of death.

2 95% Confidence interval.

# P value less than .05.

Supplementary table 6: Standardized mortality ratio (SMR) for each cause of death following esophageal cancer diagnosis in white.

Cause of Death	Less than a year		1-5 years		5-10 years		More than 10 years		Total	
	Observed[1]	SMR (95% CI[2])	Observed[1]	SMR (95% CI[2])	Observed[1]	SMR (95% CI[2])	Observed[1]	SMR (95% CI[2])	Observed[1]	SMR (95% CI[2])
All Causes of Death	27,054	24.85# (24.55-25.15)	14,236	10.44# (10.27-10.61)	1,842	2.71# (2.58-2.83)	629	1.93# (1.78-2.09)	43,761	12.65# (12.53-12.77)
All Malignant Cancer Causes of Death	24,380	92.51# (91.35-93.68)	12,379	36.83# (36.18-37.48)	1,007	6.29# (5.91-6.69)	253	3.63# (3.20-4.11)	38,019	45.83# (45.37-46.30)
Esophageal Cancer Deaths	22,090	2,629.58# (2,595.02-2,664.49)	11,054	1,009.83# (991.09-1,028.83)	699	134.65# (124.85-145.01)	137	62.75# (52.68-74.18)	33,980	1,271.63# (1,258.15-1,285.23)
Non-cancer Causes of Death										
In situ, benign or unknown behavior neoplasm	67	9.17# (7.11-11.65)	42	4.51# (3.25-6.09)	13	2.70# (1.44-4.62)	1	0.43 (0.01-2.39)	123	5.17# (4.30-6.17)

Tuberculosis	0	0.00# (0.00-19.47)	0	0.00# (0.00-16.32)	1	9.98# (0.25-55.60)	0	0.00# (0.00-85.62)	1	1.79# (0.05-9.97)
Syphilis	0	0.00# (0.00-345.08)	0	0.00# (0.00-262.63)	0	0.00# (0.00-512.55)	0	0.00# (0.00-1,172.35)	0	0.00# (0.00-105.16)
Septicemia	111	7.53# (6.20-9.07)	77	4.10# (3.24-5.13)	24	2.53# (1.62-3.76)	8	1.76# (0.76-3.46)	220	4.63# (4.04-5.28)
Other Infectious Diseases	49	5.97# (4.42-7.89)	25	2.36# (1.53-3.48)	8	1.56# (0.67-3.06)	0	0.00# (0.00-1.66)	82	3.13# (2.49-3.89)
Diabetes Mellitus	63	2.02# (1.55-2.59)	45	1.14# (0.83-1.52)	14	0.73# (0.40-1.22)	7	0.80# (0.32-1.66)	129	1.31# (1.09-1.55)
Alzheimer’s	20	0.57# (0.35-0.88)	30	0.67# (0.45-0.96)	30	1.16# (0.78-1.65)	19	1.23# (0.74-1.93)	99	0.82# (0.67-1.00)
Diseases of Heart	911	3.00# (2.81-3.21)	645	1.75# (1.62-1.89)	299	1.66# (1.48-1.86)	138	1.60# (1.34-1.89)	1,993	2.12# (2.03-2.22)
Hypertension without Heart Disease	32	3.17# (2.17-4.47)	17	1.31# (0.76-2.10)	12	1.70# (0.88-2.96)	7	1.88# (0.75-3.87)	68	2.01# (1.56-2.54)
Cerebrovascular Diseases	144	2.38# (2.01-2.80)	91	1.26# (1.01-1.54)	48	1.35# (1.00-1.79)	19	1.08# (0.65-1.68)	302	1.62# (1.45-1.82)

Atherosclerosis	19	4.53# (2.73-7.07)	9	1.97# (0.90-3.74)	2	1.01# (0.12-3.64)	1	1.15# (0.03-6.39)	31	2.67# (1.81-3.78)
Aortic Aneurysm and Dissection	18	2.82# (1.67-4.45)	13	1.73# (0.92-2.96)	2	0.62# (0.07-2.22)	2	1.49# (0.18-5.38)	35	1.89# (1.32-2.63)
Other Diseases of Arteries, Arterioles, Capillaries	11	2.59# (1.29-4.63)	10	1.92# (0.92-3.53)	4	1.56# (0.43-4.00)	3	2.47# (0.51-7.21)	28	2.11# (1.41-3.06)
Pneumonia and Influenza	102	3.83# (3.12-4.64)	65	2.04# (1.58-2.61)	33	2.11# (1.45-2.96)	13	1.71# (0.91-2.92)	213	2.61# (2.27-2.98)
Chronic Obstructive Pulmonary Disease	242	3.40# (2.99-3.86)	218	2.39# (2.08-2.73)	98	2.13# (1.73-2.59)	51	2.36# (1.76-3.11)	609	2.65# (2.44-2.87)
Stomach and Duodenal Ulcers	4	2.63# (0.72-6.73)	5	2.77# (0.90-6.47)	1	1.21# (0.03-6.73)	1	2.63# (0.07-14.64)	11	2.43# (1.21-4.34)
Chronic Liver Disease and Cirrhosis	73	6.13# (4.80-7.70)	26	1.69# (1.10-2.48)	15	2.18# (1.22-3.59)	7	2.50# (1.00-5.14)	121	3.27# (2.71-3.91)
Nephritis, Nephrotic Syndrome and Nephrosis	50	2.44# (1.81-3.22)	31	1.19# (0.81-1.69)	17	1.27# (0.74-2.03)	9	1.39# (0.63-2.64)	107	1.61# (1.32-1.95)

Complications of Pregnancy, Childbirth, Puerperium	1	319.82# (8.10-1,781.90)	0	0.00# (0.00-959.71)	0	0.00# (0.00-2,836.81)	0	0.00# (0.00-7,957.39)	1	114.49# (2.90-637.89)
Congenital Anomalies	2	2.10# (0.25-7.60)	0	0.00# (0.00-3.12)	1	1.91# (0.05-10.66)	0	0.00# (0.00-16.72)	3	1.04# (0.22-3.05)
Certain Conditions Originating in Perinatal Period	0	0.00# (0.00-957.62)	0	0.00# (0.00-741.19)	0	0.00# (0.00-1,673.12)	0	0.00# (0.00-4,159.59)	0	0.00# (0.00-309.45)
Symptoms, Signs and Ill-Defined Conditions	62	5.52# (4.23-7.08)	38	2.70# (1.91-3.71)	7	0.94# (0.38-1.93)	6	1.67# (0.61-3.64)	113	3.11# (2.56-3.74)
Accidents and Adverse Effects	53	1.69# (1.27-2.22)	61	1.53# (1.17-1.97)	34	1.71# (1.18-2.39)	10	1.04# (0.50-1.91)	158	1.57# (1.34-1.84)
Suicide and Self-Inflicted Injury	81	8.81# (7.00-10.95)	32	2.74# (1.87-3.86)	8	1.52# (0.66-3.00)	3	1.39# (0.29-4.07)	124	4.38# (3.64-5.22)
Homicide and Legal Intervention	0	0.00# (0.00-3.63)	3	2.45# (0.50-7.15)	0	0.00# (0.00-7.53)	0	0.00# (0.00-20.55)	3	1.03# (0.21-3.01)
Other Cause of Death	559	3.62# (3.32-3.93)	374	1.87# (1.69-2.07)	164	1.50# (1.28-1.75)	71	1.24 (0.97-1.57)	1168	2.24# (2.12-2.38)

1 number of cancer patients who died due to each cause of death.

2 95% Confidence interval.

# P value less than .05.

Supplementary table 7: Standardized mortality ratio (SMR) for each cause of death following esophageal cancer diagnosis in Black.

Cause of Death	Less than a year		1-5 years		5-10 years		More than 10 years		Total	
	Observed[1]	SMR (95% CI[2])	Observed[1]	SMR (95% CI[2])	Observed[1]	SMR (95% CI[2])	Observed[1]	SMR (95% CI[2])	Observed[1]	SMR (95% CI[2])
All Causes of Death	4,237	35.74# (34.67-36.83)	1,715	13.88# (13.23-14.56)	209	3.73# (3.24-4.27)	57	2.30# (1.74-2.98)	6,218	19.26# (18.78-19.74)
All Malignant Cancer Causes of Death	3,809	123.35# (119.47-127.33)	1,469	45.59# (43.29-47.98)	113	8.16# (6.73-9.82)	29	5.06# (3.39-7.27)	5,420	65.56# (63.83-67.33)
Esophageal Cancer Deaths	3,395	4,146.31# (4,008.00-4,288.18)	1,299	1,625.19# (1,538.00-1,716.03)	67	222.98# (172.81-283.18)	10	90.67# (43.48-166.75)	4,771	2,351.58# (2,285.32-2,419.27)
Non-cancer Causes of Death										
In situ, benign or unknown behavior neoplasm	5	9.70# (3.15-22.64)	4	7.27# (1.98-18.61)	2	7.69# (0.93-27.79)	0	0.00 (0.00-30.33)	11	7.60# (3.79-13.60)

Tuberculosis	0	0.00# (0.00-56.93)	0	0.00# (0.00-60.77)	0	0.00# (0.00-178.46)	0	0.00# (0.00-507.41)	0	0.00# (0.00-24.04)
Syphilis	0	0.00# (0.00-412.10)	0	0.00# (0.00-400.26)	0	0.00# (0.00-880.19)	0	0.00# (0.00-2,044.14)	0	0.00# (0.00-152.67)
Septicemia	17	6.47# (3.77-10.35)	13	4.70# (2.50-8.04)	7	5.59# (2.25-11.52)	2	3.72# (0.45-13.44)	39	5.43# (3.86-7.42)
Other Infectious Diseases	28	14.44# (9.60-20.87)	12	6.46# (3.34-11.29)	4	5.75# (1.57-14.72)	1	3.89# (0.10-21.68)	45	9.48# (6.91-12.68)
Diabetes Mellitus	8	1.44# (0.62-2.83)	6	1.03# (0.38-2.24)	2	0.77# (0.09-2.78)	0	0.00# (0.00-3.29)	16	1.06# (0.61-1.72)
Alzheimer's	3	1.47# (0.30-4.29)	2	0.86# (0.10-3.09)	2	1.36# (0.16-4.90)	0	0.00# (0.00-4.20)	7	1.04# (0.42-2.14)
Diseases of Heart	135	4.08# (3.42-4.83)	82	2.42# (1.93-3.01)	38	2.54# (1.80-3.49)	12	1.84# (0.95-3.21)	267	3.02# (2.67-3.41)
Hypertension without Heart Disease	9	4.32# (1.97-8.20)	9	4.04# (1.85-7.67)	1	0.92# (0.02-5.11)	0	0.00# (0.00-7.28)	19	3.22# (1.94-5.02)
Cerebrovascular Diseases	29	3.82# (2.56-5.49)	11	1.41# (0.70-2.53)	6	1.71# (0.63-3.71)	2	1.24# (0.15-4.48)	48	2.34# (1.73-3.10)
Atherosclerosis	1	3.08#	0	0.00#	0	0.00#	0	0.00#	1	1.21#

		(0.08-17.16)		(0.00-11.66)		(0.00-27.69)		(0.00-67.81)		(0.03-6.72)
Aortic Aneurysm and Dissection	1	2.11# (0.05-11.78)	1	2.16# (0.05-12.04)	0	0.00# (0.00-20.33)	0	0.00# (0.00-51.08)	2	1.68# (0.20-6.08)
Other Diseases of Arteries, Arterioles, Capillaries	1	1.73# (0.04-9.61)	0	0.00# (0.00-6.12)	0	0.00# (0.00-13.07)	0	0.00# (0.00-28.68)	1	0.63# (0.02-3.50)
Pneumonia and Influenza	24	9.91# (6.35-14.74)	11	4.37# (2.18-7.82)	1	0.86# (0.02-4.81)	2	3.89# (0.47-14.04)	38	5.75# (4.07-7.89)
Chronic Obstructive Pulmonary Disease	25	5.75# (3.72-8.49)	18	3.83# (2.27-6.06)	7	3.15# (1.26-6.48)	2	1.94# (0.24-7.02)	52	4.23# (3.16-5.55)
Stomach and Duodenal Ulcers	0	0.00# (0.00-24.65)	0	0.00# (0.00-25.01)	1	17.06# (0.43-95.07)	0	0.00# (0.00-157.06)	1	2.64# (0.07-14.69)
Chronic Liver Disease and Cirrhosis	7	6.48# (2.61-13.36)	3	2.83# (0.58-8.26)	0	0.00# (0.00-9.19)	0	0.00# (0.00-24.04)	10	3.71# (1.78-6.82)
Nephritis, Nephrotic Syndrome and Nephrosis	19	5.19# (3.13-8.11)	6	1.53# (0.56-3.32)	0	0.00# (0.00-20.00)	0	0.00# (0.00-4.49)	25	2.44# (1.58-3.60)
Complications of Pregnancy,	1	412.82#	0	0.00#	0	0.00#	0	0.00#	1	174.58#

Childbirth, Puerperium		(10.45-2,300.10)		(0.00-1,484.41)		(0.00-4,965.57)		(0.00-47,418.13)		(4.42-972.68)
Congenital Anomalies	2	21.98# (2.66-79.41)	1	11.09# (0.28-61.76)	0	0.00# (0.00-103.18)	0	0.00# (0.00-259.47)	3	12.98# (2.68-37.93)
Certain Conditions Originating in Perinatal Period	0	0.00# (0.00-5,672.87)	1	1,492.27# (37.78-8,314.38)	0	0.00# (0.00-14,909.39)	0	0.00# (0.00-46,244.42)	1	606.95# (15.37-3,381.72)
Symptoms, Signs and Ill-Defined Conditions	18	14.94# (8.85-23.61)	7	5.60# (2.25-11.54)	2	3.23# (0.39-11.66)	1	3.96# (0.10-22.09)	28	8.42# (5.59-12.17)
Accidents and Adverse Effects	12	4.09# (2.11-7.14)	7	2.38# (0.96-4.91)	3	2.49# (0.51-7.26)	2	3.78# (0.46-13.66)	24	3.15# (2.02-4.69)
Suicide and Self-Inflicted Injury	0	0.00# (0.00-12.56)	1	3.60# (0.09-20.04)	0	0.00# (0.00-36.06)	0	0.00# (0.00-94.12)	1	1.40# (0.04-7.81)
Homicide and Legal Intervention	0	0.00# (0.00-7.83)	3	7.18# (1.48-20.97)	0	0.00# (0.00-27.44)	0	0.00# (0.00-80.47)	3	2.80# (0.58-8.20)
Other Cause of Death	83	5.87# (4.68-7.28)	48	3.13# (2.31-4.15)	20	2.53# (1.55-3.91)	4	1.04 (0.28-2.67)	155	3.76# (3.19-4.40)

1 number of cancer patients who died due to each cause of death.

2 95% Confidence interval.

# P value less than .05.

Supplementary table 8: Standardized mortality ratio (SMR) for each cause of death following esophageal cancer diagnosis in Asian or Pacific Islander.

Cause of Death	Less than a year		1-5 years		5-10 years		More than 10 years		Total	
	Observed[1]	SMR (95% CI[2])	Observed[1]	SMR (95% CI[2])	Observed[1]	SMR (95% CI[2])	Observed[1]	SMR (95% CI[2])	Observed[1]	SMR (95% CI[2])
All Causes of Death	1,460	39.17# (37.19-41.24)	710	16.54# (15.35-17.81)	74	3.15# (2.47-3.96)	27	2.63# (1.73-3.82)	2,271	19.93# (19.12-20.77)
All Malignant Cancer Causes of Death	1,350	149.50# (141.63-157.69)	645	61.25# (56.62-66.17)	42	8.03# (5.79-10.86)	11	5.21# (2.60-9.32)	2,048	76.13# (72.87-79.50)
Esophageal Cancer Deaths	1,240	6,367.89# (6,018.35-6,732.44)	568	2,574.12# (2,366.76-2,794.79)	27	270.19# (178.05-393.11)	9	247.81# (113.32-470.42)	1,844	3,342.80# (3,191.95-3,498.93)
Non-cancer Causes of Death										
In situ, benign or unknown behavior neoplasm	1	4.29 (0.11-23.93)	1	3.65 (0.09-20.35)	0	0.00 (0.00-23.74)	0	0.00 (0.00-54.92)	2	2.74 (0.33-9.91)

Tuberculosis	1	13.91# (0.35-77.50)	0	0.00# (0.00-45.98)	0	0.00# (0.00-87.54)	0	0.00# (0.00-226.39)	1	4.75# (0.12-26.46)
Syphilis	0	0.00# (0.00-5,775.31)	0	0.00# (0.00-5,606.74)	0	0.00# (0.00-10,977. 38)	0	0.00# (0.00-24,661. 76)	0	0.00# (0.00-2,069. 74)
Septicemia	8	16.52# (7.13-32.55)	2	3.54# (0.43-12.77)	0	0.00# (0.00-12.01)	2	15.27# (1.85-55.16)	12	8.06# (4.17-14.09)
Other Infectious Diseases	2	5.60# (0.68-20.25)	1	2.41# (0.06-13.43)	0	0.00# (0.00-17.90)	0	0.00# (0.00-45.34)	3	2.83# (0.58-8.28)
Diabetes Mellitus	4	2.41# (0.66-6.18)	0	0.00# (0.00-1.90)	0	0.00# (0.00-3.62)	0	0.00# (0.00-8.46)	4	0.79# (0.22-2.03)
Alzheimer’s	1	1.11# (0.03-6.18)	0	0.00# (0.00-3.30)	0	0.00# (0.00-4.53)	0	0.00# (0.00-8.00)	1	0.30# (0.01-1.69)
Diseases of Heart	32	3.29# (2.25-4.65)	27	2.48# (1.63-3.60)	11	1.87# (0.93-3.34)	4	1.57# (0.43-4.03)	74	2.55# (2.00-3.20)
Hypertension without Heart Disease	2	3.39# (0.41-12.26)	0	0.00# (0.00-5.23)	0	0.00# (0.00-8.31)	0	0.00# (0.00-16.93)	2	1.02# (0.12-3.69)
Cerebrovascular Diseases	7	2.40# (0.97-4.95)	4	1.21# (0.33-3.10)	4	2.25# (0.61-5.75)	1	1.26# (0.03-7.04)	16	1.82# (1.04-2.96)
Atherosclerosis	0	0.00#	0	0.00#	0	0.00#	0	0.00#	0	0.00#

		(0.00-34.79)		(0.00-32.93)		(0.00-61.98)		(0.00-147.84)		(0.00-12.19)
Aortic Aneurysm and Dissection	1	4.23# (0.11-23.57)	1	3.83# (0.10-21.34)	0	0.00# (0.00-30.07)	0	0.00# (0.00-80.84)	2	3.00# (0.36-10.85)
Other Diseases of Arteries, Arterioles, Capillaries	0	0.00# (0.00-33.74)	0	0.00# (0.00-29.67)	0	0.00# (0.00-55.24)	0	0.00# (0.00-128.19)	0	0.00# (0.00-11.21)
Pneumonia and Influenza	4	2.84# (0.77-7.28)	5	3.18# (1.03-7.41)	4	4.20# (1.14-10.74)	2	4.62# (0.56-16.70)	15	3.43# (1.92-5.67)
Chronic Obstructive Pulmonary Disease	6	3.61# (1.33-7.86)	6	3.18# (1.17-6.91)	2	1.85# (0.22-6.67)	2	4.26# (0.52-15.38)	16	3.14# (1.79-5.09)
Stomach and Duodenal Ulcers	2	23.29# (2.82-84.12)	0	0.00# (0.00-39.21)	0	0.00# (0.00-74.84)	0	0.00# (0.00-182.73)	2	8.02# (0.97-28.96)
Chronic Liver Disease and Cirrhosis	2	5.30# (0.64-19.14)	1	2.32# (0.06-12.91)	0	0.00# (0.00-19.68)	0	0.00# (0.00-52.06)	3	2.81# (0.58-8.21)
Nephritis, Nephrotic Syndrome and Nephrosis	0	0.00# (0.00-4.47)	1	1.03# (0.03-5.75)	1	1.80# (0.05-10.02)	0	0.00# (0.00-15.05)	2	0.77# (0.09-2.78)
Complications of Pregnancy,	0	0.00#	0	0.00#	0	0.00#	0	0.00#	0	0.00#

Childbirth, Puerperium		(0.00-23,380.46)		(0.00-17,863.83)		(0.00-44,035.08)		(0.00-108,633.24)		(0.00-7,653.20)
Congenital Anomalies	0	0.00# (0.00-144.33)	0	0.00# (0.00-127.62)	0	0.00# (0.00-264.43)	0	0.00# (0.00-621.22)	0	0.00# (0.00-49.61)
Certain Conditions Originating in Perinatal Period	0	0.00# (0.00-19,430.80)	0	0.00# (0.00-21,838.80)	0	0.00# (0.00-51,198.17)	0	0.00# (0.00-55,815.45)	0	0.00# (0.00-7,423.75)
Symptoms, Signs and Ill-Defined Conditions	1	3.57# (0.09-19.92)	2	6.13# (0.74-22.13)	0	0.00# (0.00-18.61)	1	11.57# (0.29-64.48)	4	4.49# (1.22-11.50)
Accidents and Adverse Effects	1	0.91# (0.02-5.08)	2	1.60# (0.19-5.77)	1	1.50# (0.04-8.33)	0	0.00# (0.00-12.60)	4	1.21# (0.33-3.09)
Suicide and Self-Inflicted Injury	6	28.79# (10.57-62.66)	0	0.00# (0.00-16.12)	0	0.00# (0.00-38.47)	1	28.37# (0.72-158.08)	7	12.31# (4.95-25.37)
Homicide and Legal Intervention	0	0.00# (0.00-68.81)	0	0.00# (0.00-65.00)	0	0.00# (0.00-179.11)	0	0.00# (0.00-508.45)	0	0.00# (0.00-26.69)
Other Cause of Death	29	6.00# (4.02-8.62)	12	2.09# (1.08-3.66)	9	2.55# (1.17-4.85)	3	1.80 (0.37-5.27)	53	3.36# (2.52-4.40)

1 number of cancer patients who died due to each cause of death.

2 95% Confidence interval.

# P value less than .05.

Supplementary table 9: Standardized mortality ratio (SMR) for each cause of death following esophageal cancer diagnosis in Indian American or Alaska Native.

Cause of Death	Less than a year		1-5 years		5-10 years		More than 10 years		Total	
	Observed[1]	SMR (95% CI[2])	Observed[1]	SMR (95% CI[2])	Observed[1]	SMR (95% CI[2])	Observed[1]	SMR (95% CI[2])	Observed[1]	SMR (95% CI[2])
All Causes of Death	172	66.11# (56.60-76.76)	70	24.30# (18.94-30.7)	9	5.16# (2.36-9.80)	2	2.81 (0.34-10.17)	253	31.88# (28.07-36.06)
All Malignant Cancer Causes of Death	157	232.15# (197.26-271.44)	65	85.13# (65.70-108.51)	8	19.06# (8.23-37.55)	0	0.00 (0.00-22.78)	230	113.78# (99.55-129.47)
Esophageal Cancer Deaths	148	9,424.93# (7,967.69-11,071.56)	59	3,139.20# (2,389.71-4,049.34)	7	729.93# (293.47-1,503.93)	0	0.00 (0.00-1,192.01)	214	4,535.60# (3,948.24-5,185.72)
Non-cancer Causes of Death										
In situ, benign or unknown behavior neoplasm	2	125.10# (15.15-451.91)	0	0.00 (0.00-205.36)	0	0.00 (0.93-319.23)	0	0.00 (0.00-757.04)	2	39.70# (4.81-143.42)

Tuberculosis	0	0.00 (0.00-718.76)	0	0.00 (0.00-643.99)	0	0.00 (0.00-1,101.21)	0	0.00 (0.00-2,810.59)	0	0.00 (0.00-237.64)
Syphilis	0	0.00 (0.00-91,972.54)	0	0.00 (0.00-69,842.65)	0	0.00 (0.00-149,241.23)	0	0.00 (0.00-321,184.35)	0	0.00 (0.00-28,567.55)
Septicemia	0	0.00 (0.00-110.12)	0	0.00 (0.00-98.14)	0	0.00 (0.00-161.24)	0	0.00 (0.00-396.21)	0	0.00 (0.00-35.72)
Other Infectious Diseases	0	0.00 (0.00-129.37)	0	0.00 (0.00-119.44)	0	0.00 (0.00-237.45)	0	0.00 (0.00-625.66)	0	0.00 (0.00-45.64)
Diabetes Mellitus	1	8.31 (0.21-46.32)	1	7.52 (0.19-41.90)	0	0.00 (0.00-47.66)	0	0.00 (0.00-117.49)	2	5.52 (0.67-19.96)
Alzheimer’s	0	0.00 (0.00-80.2)	0	0.00 (0.00-81.73)	0	0.00 (0.00-83.52)	0	0.00 (0.00-148.44)	0	0.00 (0.00-23.03)
Diseases of Heart	3	4.50 (0.93-13.14)	1	1.36 (0.03-7.56)	1	2.25 (0.06-12.56)	0	0.00 (0.00-21.20)	5	2.47 (0.80-5.77)
Hypertension without Heart Disease	0	0.00 (0.00-100.42)	0	0.00 (0.00-92.31)	0	0.00 (0.00-127.17)	0	0.00 (0.00-275.91)	0	0.00 (0.00-30.98)
Cerebrovascular Diseases	0	0.00 (0.00-19.30)	0	0.00 (0.00-18.43)	0	0.00 (0.00-30.17)	0	0.00 (0.00-71.02)	0	0.00 (0.00-6.52)

Atherosclerosis	0	0.00 (0.00-543.39)	0	0.00 (0.00-516.54)	0	0.00 (0.00-951.68)	0	0.00 (0.00-2,423.57)	0	0.00 (0.00-190.85)
Aortic Aneurysm and Dissection	0	0.00 (0.00-226.23)	0	0.00 (0.00-208.66)	0	0.00 (0.00-384.38)	0	0.00 (0.00-1,123.80)	0	0.00 (0.00-78.72)
Other Diseases of Arteries, Arterioles, Capillaries	0	0.00 (0.00-487.32)	0	0.00 (0.00-459.82)	0	0.00 (0.00-811.13)	0	0.00 (0.00-2,007.26)	0	0.00 (0.00-167.85)
Pneumonia and Influenza	0	0.00 (0.00-41.00)	0	0.00 (0.00-35.76)	0	0.00 (0.00-52.19)	0	0.00 (0.00-128.98)	0	0.00 (0.00-12.61)
Chronic Obstructive Pulmonary Disease	0	0.00 (0.00-33.25)	1	7.87 (0.20-43.82)	0	0.00 (0.00-43.95)	0	0.00 (0.00-104.59)	1	2.80 (0.07-15.59)
Stomach and Duodenal Ulcers	0	0.00 (0.00-650.63)	0	0.00 (0.00-584.35)	0	0.00 (0.00-972.42)	0	0.00 (0.00-2,665.59)	0	0.00 (0.00-214.97)
Chronic Liver Disease and Cirrhosis	2	57.13# (6.92-206.38)	1	25.91 (0.66-144.37)	0	0.00 (0.00-226.66)	0	0.00 (0.00-780.72)	3	31.71# (6.54-92.68)

Nephritis, Nephrotic Syndrome and Nephrosis	1	17.99 (0.46-100.21)	0	0.00 (0.00-58.82)	0	0.00 (0.00-90.51)	1	58.45# (1.48-325.69)	2	11.35# (1.37-41.01)
Complications of Pregnancy, Childbirth, Puerperium	0	0.00 (0.00-238,732.48)	0	0.00 (0.00-199,238.77)	0	0.00 (0.00-1,176,611.46)	0	0.00 (0.00-0.00)	0	0.00 (0.00-99,425.41)
Congenital Anomalies	0	0.00 (0.00-1,756.93)	0	0.00 (0.00-1,622.23)	0	0.00 (0.00-3,238.10)	0	0.00 (0.00-8,826.97)	0	0.00 (0.00-622.00)
Certain Conditions Originating in Perinatal Period	0	0.00 (0.00-315,183.18)	0	0.00 (0.00-361,164.84)	0	0.00 (0.00-382,421.32)	0	0.00 (0.00-579,104.24)	0	0.00 (0.00-97,245.06)
Symptoms, Signs and Ill-Defined Conditions	0	0.00 (0.00-196.48)	0	0.00 (0.00-179.88)	0	0.00 (0.00-275.79)	0	0.00 (0.00-785.9)	0	0.00 (0.00-64.32)
Accidents and Adverse Effects	1	11.59 (0.29-64.59)	0	0.00 (0.00-38.71)	0	0.00 (0.00-69.56)	0	0.00 (0.00-176.55)	1	3.91 (0.10-21.81)
Suicide and Self-Inflicted Injury	2	96.41# (11.68-348.27)	0	0.00 (0.00-161.94)	0	0.00 (0.00-390.06)	0	0.00 (0.00-1,408.74)	2	35.97# (4.36-129.94)

Homicide and Legal Intervention	0	0.00 (0.00-631.52)	0	0.00 (0.00-644.21)	0	0.00 (0.00-1,904.95)	0	0.00 (0.00-9,537.73)	0	0.00 (0.00-265.56)
Other Cause of Death	3	9.40# (1.94-27.47)	1	2.84 (0.07-15.82)	0	0.00 (0.00-15.32)	1	9.18 (0.23-51.15)	5	4.90# (1.59-11.43)

1 number of cancer patients who died due to each cause of death.

2 95% Confidence interval.

# P value less than .05.

Supplementary table 10: Standardized mortality ratio (SMR) for each cause of death following localized esophageal cancer diagnosis.

Cause of Death	Less than a year		1-5 years		5-10 years		More than 10 years		Total	
	Observed[1]	SMR (95% CI[2])	Observed[1]	SMR (95% CI[2])	Observed[1]	SMR (95% CI[2])	Observed[1]	SMR (95% CI[2])	Observed[1]	SMR (95% CI[2])
All Causes of Death	3,117	11.74# (11.33-12.16)	2,543	5.02# (4.83-5.22)	631	2.26# (2.09-2.44)	134	1.71# (1.43-2.03)	6,425	5.69# (5.55-5.83)
All Malignant Cancer Causes of Death	2,516	40.41# (38.85-42.02)	1,907	15.67# (14.98-16.39)	306	4.79# (4.27-5.36)	49	3.05# (2.25-4.03)	4,778	18.11# (17.60-18.63)
Esophageal	2,292	1,209.09#	1,664	430.38#	207	101.68#	24	48.78#	4,187	505.08#

Cancer Deaths		(1,160.09-1,259.63)		(409.94-451.56)		(88.30-116.51)		(31.26-72.58)		(489.89-520.61)
Non-cancer Causes of Death										
In situ, benign or unknown behavior neoplasm	10	5.59# (2.68-10.29)	10	2.87# (1.37-5.27)	9	4.58# (2.09-8.69)	0	0.00 (0.00-6.79)	29	3.72# (2.49-5.35)
Tuberculosis	1	16.66 (0.42-92.80)	0	0.00 (0.00-34.25)	0	0.00 (0.00-67.01)	0	0.00 (0.00-239.69)	1	4.20 (0.11-23.39)
Syphilis	0	0.00 (0.00-935.18)	0	0.00 (0.00-510.49)	0	0.00 (0.00-941.43)	0	0.00 (0.00-3,788.62)	0	0.00 (0.00-229.65)
Septicemia	24	6.28# (4.03-9.35)	24	3.29# (2.11-4.90)	9	2.24# (1.02-4.25)	1	0.89 (0.02-4.98)	58	3.57# (2.71-4.62)
Other Infectious Diseases	9	4.15# (1.90-7.89)	11	2.65# (1.32-4.74)	4	1.89 (0.51-4.83)	0	0.00 (0.00-7.05)	24	2.68# (1.72-3.99)
Diabetes Mellitus	18	2.28# (1.35-3.60)	22	1.45 (0.91-2.19)	8	0.98 (0.42-1.93)	2	0.92 (0.11-3.31)	50	1.49# (1.11-1.97)
Alzheimer’s	6	0.63 (0.23-1.38)	10	0.56 (0.27-1.03)	13	1.16 (0.62-1.99)	11	2.76# (1.38-4.93)	40	0.94 (0.67-1.28)
Diseases of Heart	196	2.71# (2.34-3.11)	222	1.65# (1.44-1.88)	113	1.54# (1.27-1.86)	21	1.01 (0.62-1.54)	552	1.84# (1.69-1.99)

Hypertension without Heart Disease	11	3.80# (1.90-6.81)	9	1.64 (0.75-3.12)	6	1.86 (0.68-4.05)	2	2.00 (0.24-7.22)	28	2.22# (1.48-3.21)
Cerebrovascular Diseases	37	2.50# (1.76-3.45)	26	0.97 (0.63-1.42)	17	1.15 (0.67-1.84)	7	1.58 (0.64-3.26)	87	1.43# (1.14-1.76)
Atherosclerosis	0	0.00 (0.00-4.11)	0	0.00 (0.00-2.48)	0	0.00 (0.00-5.00)	1	5.12 (0.13-28.50)	1	0.30 (0.01-1.68)
Aortic Aneurysm and Dissection	3	2.18 (0.45-6.36)	2	0.80 (0.10-2.90)	0	0.00 (0.00-3.07)	2	6.59 (0.80-23.81)	7	1.30 (0.52-2.68)
Other Diseases of Arteries, Arterioles, Capillaries	3	2.88 (0.59-8.43)	3	1.55 (0.32-4.54)	1	0.95 (0.02-5.32)	0	0.00 (0.00-12.56)	7	1.62 (0.65-3.35)
Pneumonia and Influenza	22	3.38# (2.12-5.12)	17	1.44 (0.84-2.31)	10	1.55 (0.74-2.85)	4	2.11 (0.58-5.41)	53	1.99# (1.49-2.60)
Chronic Obstructive Pulmonary Disease	53	3.14# (2.35-4.11)	89	2.69# (2.16-3.31)	32	1.75# (1.20-2.48)	10	2.03 (0.97-3.73)	184	2.52# (2.17-2.91)
Stomach and Duodenal Ulcers	3	8.71# (1.80-25.46)	1	1.58 (0.04-8.80)	0	0.00 (0.00-11.02)	1	10.79 (0.27-60.10)	5	3.56# (1.16-8.31)
Chronic Liver Disease and	24	9.43#	9	1.72	4	1.48	2	3.07	39	3.50#

Cirrhosis		(6.04-14.03)		(0.79-3.26)		(0.40-3.78)		(0.37-11.08)		(2.49-4.78)
Nephritis, Nephrotic Syndrome and Nephrosis	18	3.25# (1.92-5.13)	20	1.92# (1.17-2.96)	8	1.39 (0.60-2.74)	0	0.00 (0.00-2.29)	46	1.97# (1.44-2.63)
Complications of Pregnancy, Childbirth, Puerperium	0	0.00 (0.00-3,758.22)	0	0.00 (0.00-2,215.97)	0	0.00 (0.00-10,917.95)	0	0.00 (0.00-264,116.3)	0	0.00 (0.00-1,230.42)
Congenital Anomalies	0	0.00 (0.00-18.06)	0	0.00 (0.00-9.27)	0	0.00 (0.00-18.12)	0	0.00 (0.00-70.41)	0	0.00 (0.00-4.30)
Certain Conditions Originating in Perinatal Period	0	0.00 (0.00-4,064.60)	1	563.99# (14.28-3,142.34)	0	0.00 (0.00-4,202.38)	0	0.00 (0.00-14,686.09)	1	262.49# (6.65-1,462.51)
Symptoms, Signs and Ill-Defined Conditions	14	4.75# (2.60-7.96)	13	2.38# (1.27-4.08)	2	0.65 (0.08-2.36)	2	2.51 (0.30-9.07)	31	2.53# (1.72-3.59)
Accidents and Adverse Effects	8	1.07 (0.46-2.11)	17	1.16 (0.67-1.85)	18	2.20# (1.30-3.47)	1	0.43 (0.01-2.38)	44	1.35 (0.98-1.81)
Suicide and Self-Inflicted Injury	12	6.40# (3.31-11.19)	9	2.32# (1.06-4.41)	5	2.48 (0.81-5.79)	1	2.06 (0.05-11.49)	27	3.27# (2.16-4.76)
Homicide and Legal Intervention	0	0.00	1	2.17	0	0.00	0	0.00	1	1.03

		(0.00-14.67)		(0.05-12.08)		(0.00-17.58)		(0.00-78.74)		(0.03-5.75)
Other Cause of Death	129	3.22# (2.69-3.82)	120	1.53# (1.27-1.83)	66	1.42# (1.10-1.80)	17	1.22 (0.71-1.96)	332	1.86# (1.66-2.07)

1 number of cancer patients who died due to each cause of death.

2 95% Confidence interval.

# P value less than .05.

Supplementary table 11: Standardized mortality ratio (SMR) for each cause of death following regional esophageal cancer diagnosis.

Cause of Death	Less than a year		1-5 years		5-10 years		More than 10 years		Total	
	Observed[1]	SMR (95% CI[2])	Observed[1]	SMR (95% CI[2])	Observed[1]	SMR (95% CI[2])	Observed[1]	SMR (95% CI[2])	Observed[1]	SMR (95% CI[2])
All Causes of Death	5,898	17.89# (17.44-18.35)	5,308	12.63# (12.29-12.98)	588	3.41# (3.14-3.70)	95	2.77# (2.24-3.38)	11,889	12.43# (12.20-12.65)
All Malignant Cancer Causes of Death	5,196	62.01# (60.33-63.72)	4,691	43.06# (41.83-44.31)	367	8.72# (7.85-9.66)	48	5.86# (4.32-7.77)	10,302	42.39# (41.58-43.22)
Esophageal Cancer Deaths	4,820	1,755.52# (1,706.3-1,805.79)	4,212	1,149.15# (1,114.71-1,184.39)	262	191.61# (169.11-216.28)	30	111.27# (75.07-158.84)	9,324	1,158.56# (1,135.16-1,182.32)

Non-cancer Causes of Death										
In situ, benign or unknown behavior neoplasm	12	5.51# (2.85-9.63)	17	6.06# (3.53-9.71)	1	0.84 (0.02-4.69)	0	0.00 (0.00-15.21)	30	4.68# (3.16-6.68)
Tuberculosis	0	0.00 (0.00-45.35)	0	0.00 (0.00-38.07)	0	0.00 (0.00-99.19)	0	0.00 (0.00-569.13)	0	0.00 (0.00-16.62)
Syphilis	0	0.00 (0.00-680.59)	0	0.00 (0.00-536.86)	0	0.00 (0.00-1,287.75)	0	0.00 (0.00-8,066.15)	0	0.00 (0.00-236.27)
Septicemia	48	10.00# (7.37-13.25)	26	4.22# (2.76-6.19)	8	3.15# (1.36-6.20)	1	1.97 (0.05-10.95)	83	5.92# (4.72-7.34)
Other Infectious Diseases	21	6.85# (4.24-10.47)	13	3.33# (1.77-5.70)	3	2.12 (0.44-6.19)	1	3.86 (0.10-21.53)	38	4.40# (3.11-6.03)
Diabetes Mellitus	9	0.86 (0.39-1.63)	10	0.74 (0.35-1.36)	5	0.93 (0.30-2.17)	1	0.94 (0.02-5.25)	25	0.82 (0.53-1.21)
Alzheimer’s	3	0.30# (0.06-0.89)	8	0.65 (0.28-1.28)	7	1.15 (0.46-2.38)	0	0.00 (0.00-2.83)	18	0.61# (0.36-0.96)
Diseases of Heart	199	2.30# (1.99-2.64)	205	1.91# (1.65-2.18)	75	1.70# (1.34-2.13)	18	2.05# (1.22-3.24)	497	2.01# (1.84-2.20)
Hypertension without Heart	6	1.73 (0.64-3.78)	9	2.04 (0.93-3.87)	2	1.02 (0.12-3.69)	1	2.57 (0.07-14.31)	18	1.76# (1.04-2.78)

Disease										
Cerebrovascular Diseases	26	1.52 (0.99-2.23)	20	0.96 (0.58-1.48)	9	1.02 (0.47-1.94)	1	0.57 (0.01-3.19)	56	1.15 (0.87-1.50)
Atherosclerosis	0	0.00 (0.00-3.98)	3	2.88 (0.59-8.41)	1	2.46 (0.06-13.68)	0	0.00 (0.00-50.83)	4	1.63 (0.44-4.18)
Aortic Aneurysm and Dissection	5	2.95 (0.96-6.89)	3	1.47 (0.30-4.30)	1	1.34 (0.03-7.48)	0	0.00 (0.00-25.98)	9	1.95 (0.89-3.70)
Other Diseases of Arteries, Arterioles, Capillaries	4	3.22 (0.88-8.25)	2	1.29 (0.16-4.65)	2	3.12 (0.38-11.27)	0	0.00 (0.00-29.24)	8	2.25 (0.97-4.42)
Pneumonia and Influenza	34	4.66# (3.23-6.51)	22	2.48# (1.56-3.76)	8	2.12 (0.91-4.17)	3	4.03 (0.83-11.76)	67	3.24# (2.51-4.12)
Chronic Obstructive Pulmonary Disease	58	2.76# (2.09-3.56)	68	2.49# (1.93-3.15)	21	1.85# (1.15-2.83)	6	2.59 (0.95-5.64)	153	2.47# (2.09-2.89)
Stomach and Duodenal Ulcers	0	0.00 (0.00-8.67)	0	0.00 (0.00-7.02)	2	9.59# (1.16-34.63)	0	0.00 (0.00-89.56)	2	1.67 (0.20-6.01)
Chronic Liver Disease and Cirrhosis	19	4.63# (2.79-7.23)	14	2.52# (1.38-4.22)	5	2.53 (0.82-5.90)	0	0.00 (0.00-9.56)	38	3.16# (2.24-4.34)

Nephritis, Nephrotic Syndrome and Nephrosis	15	2.26# (1.26-3.73)	7	0.84 (0.34-1.73)	2	0.57 (0.07-2.05)	1	1.44 (0.04-8.02)	25	1.30 (0.84-1.92)
Complications of Pregnancy, Childbirth, Puerperium	0	0.00 (0.00-1,745.65)	0	0.00 (0.00-1,344.21)	0	0.00 (0.00-5,128.2 7)	0	0.00 (0.00-40,947. 04)	0	0.00 (0.00-650.96)
Congenital Anomalies	0	0.00 (0.00-12.19)	0	0.00 (0.00-9.29)	0	0.00 (0.00-25.83)	0	0.00 (0.00-134.89)	0	0.00 (0.00-4.24)
Certain Conditions Originating in Perinatal Period	0	0.00 (0.00-2,583.77)	0	0.00 (0.00-1,934.81)	0	0.00 (0.00-5,713.7 5)	0	0.00 (0.00-32,548. 60)	0	0.00 (0.00-901.21)
Symptoms, Signs and Ill-Defined Conditions	17	5.18# (3.01-8.29)	16	3.89# (2.23-6.32)	3	1.74 (0.36-5.10)	1	3.28 (0.08-18.26)	37	3.93# (2.77-5.41)
Accidents and Adverse Effects	17	1.67 (0.97-2.67)	20	1.51 (0.92-2.33)	7	1.33 (0.54-2.74)	3	2.80 (0.58-8.17)	47	1.58# (1.16-2.10)
Suicide and Self-Inflicted Injury	28	9.46# (6.28-13.67)	11	2.76# (1.38-4.94)	2	1.41 (0.17-5.11)	0	0.00 (0.00-13.29)	41	4.75# (3.41-6.44)
Homicide and Legal Intervention	0	0.00 (0.00-8.28)	3	5.47# (1.13-15.99)	0	0.00 (0.00-21.79)	0	0.00 (0.00-125.3)	3	2.51 (0.52-7.35)

Other Cause of Death	181	3.79# (3.26-4.38)	140	2.26# (1.90-2.67)	57	2.07# (1.57-2.68)	10	1.77 (0.85-3.26)	388	2.72 (2.45-3.00)

1 number of cancer patients who died due to each cause of death.

2 95% Confidence interval.

# P value less than .05.

Supplementary table 12: Standardized-mortality ratio (SMR) for each cause of death following distant esophageal cancer diagnosis.

Cause of Death	Less than a year		1-5 years		5-10 years		More than 10 years		Total	
	Observed[1]	SMR (95% CI[2])	Observed[1]	SMR (95% CI[2])	Observed[1]	SMR (95% CI[2])	Observed[1]	SMR (95% CI[2])	Observed[1]	SMR (95% CI[2])
All Causes of Death	12,876	54.49# (53.56-55.44)	4,096	30.21# (29.30-31.15)	126	3.79# (3.16-4.51)	21	3.51# (2.17-5.36)	17,119	41.64# (41.02-42.27)
All Malignant Cancer Causes of Death	12,197	197.62# (194.13-201.16)	3,844	109.09# (105.67-112.60)	75	9.22# (7.25-11.56)	7	4.86# (1.95-10.01)	16,123	151.35# (149.02-153.70)
Esophageal Cancer Deaths	10,867	5,149.37# (5,053.00-5,247.11)	3,431	2,888.65# (2,792.79-2,986.96)	56	210.38# (158.92-273.19)	5	106.98# (34.74-249.65)	14,359	3,976.42# (3,911.64-4,042.00)

Non-cancer Causes of Death										
In situ, benign or unknown behavior neoplasm	22	14.36# (9.00-21.74)	8	9.08# (3.92-17.89)	0	0.00 (0.00-17.12)	1	26.19 (0.66-145.95)	31	11.63# (7.90-16.51)
Tuberculosis	0	0.00 (0.00-58.80)	0	0.00 (0.00-107.03)	0	0.00 (0.00-486.19)	0	0.00 (0.00-3,608.98)	0	0.00 (0.00-34.86)
Syphilis	0	0.00 (0.00-884.56)	0	0.00 (0.00-1,636.26)	0	0.00 (0.00-6,363.46)	0	0.00 (0.00-35,355.76)	0	0.00 (0.00-518.92)
Septicemia	29	8.43# (5.65-12.11)	13	6.53# (3.48-11.16)	2	4.04 (0.49-14.60)	1	10.90 (0.28-60.73)	45	7.48# (5.45-10.00)
Other Infectious Diseases	22	9.20# (5.77-13.94)	5	3.76# (1.22-8.78)	2	6.78 (0.82-24.51)	0	0.00 (0.00-74.30)	29	7.14# (4.78-10.25)
Diabetes Mellitus	18	2.33# (1.38-3.69)	7	1.58 (0.63-3.25)	0	0.00 (0.00-3.47)	1	5.14 (0.13-28.64)	26	1.94# (1.27-2.84)
Alzheimer’s	3	0.48 (0.10-1.40)	4	1.03 (0.28-2.63)	0	0.00 (0.00-3.01)	1	4.11 (0.10-22.92)	8	0.69 (0.30-1.35)
Diseases of Heart	219	3.56# (3.10-4.06)	76	2.21# (1.74-2.76)	21	2.51# (1.55-3.84)	4	2.71 (0.74-6.93)	320	3.02# (2.70-3.37)
Hypertension	5	2.05	2	1.38	1	2.53	1	13.46	9	2.07

without Heart Disease		(0.67-4.79)		(0.17-4.97)		(0.06-14.11)		(0.34-75.02)		(0.94-3.92)
Cerebrovascular Diseases	41	3.47# (2.49-4.71)	11	1.64 (0.82-2.93)	3	1.73 (0.36-5.05)	1	3.11 (0.08-17.34)	56	2.72# (2.05-3.53)
Atherosclerosis	3	4.81 (0.99-14.06)	2	6.18 (0.75-22.32)	0	0.00 (0.00-49.07)	0	0.00 (0.00-299.63)	5	4.83# (1.57-11.28)
Aortic Aneurysm and Dissection	2	1.63 (0.20-5.90)	1	1.54 (0.04-8.61)	1	7.18 (0.18-39.98)	0	0.00 (0.00-155.89)	4	1.97 (0.54-5.03)
Other Diseases of Arteries, Arterioles, Capillaries	2	2.29 (0.28-8.26)	1	2.02 (0.05-11.26)	0	0.00 (0.00-29.78)	0	0.00 (0.00-159.05)	3	1.98 (0.41-5.78)
Pneumonia and Influenza	23	4.59# (2.91-6.88)	20	7.10# (4.34-10.97)	0	0.00 (0.00-5.32)	1	8.49 (0.22-47.33)	44	5.09# (3.70-6.84)
Chronic Obstructive Pulmonary Disease	47	3.18# (2.34-4.23)	16	1.86# (1.06-3.02)	8	3.77# (1.63-7.43)	1	2.55 (0.06-14.22)	72	2.78# (2.18-3.50)
Stomach and Duodenal Ulcers	0	0.00 (0.00-12.05)	3	17.52# (3.61-51.20)	0	0.00 (0.00-90.84)	0	0.00 (0.00-512.55)	3	5.71# (1.18-16.70)
Chronic Liver Disease and Cirrhosis	18	5.39# (3.19-8.52)	3	1.54 (0.32-4.51)	1	2.29 (0.06-12.77)	0	0.00 (0.00-48.22)	22	3.80# (2.38-5.75)

Nephritis, Nephrotic Syndrome and Nephrosis	10	2.13# (1.02-3.92)	2	0.75 (0.09-2.71)	1	1.53 (0.04-8.54)	0	0.00 (0.00-31.16)	13	1.60 (0.85-2.73)
Complications of Pregnancy, Childbirth, Puerperium	1	611.86# (15.49-3,409.04)	0	0.00 (0.00-5,494.30)	0	0.00 (0.00-34,887.04)	0	0.00 (0.00-10,798,664.78)	1	414.62# (10.50-2,310.11)
Congenital Anomalies	1	4.17 (0.11-23.23)	1	7.26 (0.18-40.46)	0	0.00 (0.00-118.49)	0	0.00 (0.00-679.91)	2	4.83 (0.58-17.45)
Certain Conditions Originating in Perinatal Period	0	0.00 (0.00-3,069.41)	0	0.00 (0.00-5,520.07)	0	0.00 (0.00-24,983.23)	0	0.00 (0.00-174,224.74)	0	0.00 (0.00-1,809.24)
Symptoms, Signs and Ill-Defined Conditions	26	11.22# (7.33-16.44)	7	5.29# (2.13-10.89)	0	0.00 (0.00-11.09)	1	18.05 (0.46-100.58)	34	8.44# (5.84-11.79)
Accidents and Adverse Effects	19	2.43# (1.47-3.80)	8	1.78 (0.77-3.50)	2	1.87 (0.23-6.76)	0	0.00 (0.00-19.40)	29	2.14# (1.43-3.07)
Suicide and Self-Inflicted Injury	19	7.69# (4.63-12.01)	5	3.57# (1.16-8.32)	1	3.33 (0.08-18.55)	0	0.00 (0.00-72.47)	25	5.92# (3.83-8.74)
Homicide and Legal Intervention	0	0.00 (0.00-9.38)	2	9.94# (1.20-35.92)	0	0.00 (0.00-95.80)	0	0.00 (0.00-564.51)	2	3.13 (0.38-11.30)

Other Cause of Death	149	4.48# (3.79-5.26)	55	2.77# (2.08-3.60)	8	1.51 (0.65-2.98)	1	1.03 (0.03-5.73)	213	3.59# (3.12-4.10)

1 number of cancer patients who died due to each cause of death.

2 95% Confidence interval.

# P value less than .05.

Supplementary table 13: Standardized mortality ratio (SMR) for each cause of death following esophageal cancer diagnosis in Grade I.

Cause of Death	Less than a year		1-5 years		5-10 years		More than 10 years		Total	
	Observed[1]	SMR (95% CI[2])	Observed[1]	SMR (95% CI[2])	Observed[1]	SMR (95% CI[2])	Observed[1]	SMR (95% CI[2])	Observed[1]	SMR (95% CI[2])
All Causes of Death	1,035	16.38# (15.40-17.41)	765	6.34# (5.90-6.81)	177	2.50# (2.15-2.90)	64	1.82# (1.40-2.32)	2,041	7.05# (6.74-7.36)
All Malignant Cancer Causes of Death	885	56.89# (53.20-60.76)	616	20.67# (19.07-22.37)	93	5.64# (4.56-6.91)	32	4.37# (2.99-6.17)	1,626	23.51# (22.38-24.68)
Esophageal Cancer Deaths	800	1,663.19# (1,549.92-1,782.55)	548	573.50# (526.48-623.59)	62	119.12# (91.33-152.71)	19	88.03# (53.00-137.46)	1,429	657.65# (623.99-692.66)

Non-cancer Causes of Death										
In situ, benign or unknown behavior neoplasm	4	9.67# (2.63-24.75)	2	2.46 (0.30-8.89)	0	0.00 (0.00-7.49)	0	0.00 (0.00-15.07)	6	3.06# (1.12-6.65)
Tuberculosis	0	0.00 (0.00-229.65)	0	0.00 (0.00-135.85)	0	0.00 (0.00-251.55)	0	0.00 (0.00-661.15)	0	0.00 (0.00-58.13)
Syphilis	0	0.00 (0.00-3,866.92)	0	0.00 (0.00-2,130.41)	0	0.00 (0.00-3,461.70)	0	0.00 (0.00-7,231.86)	0	0.00 (0.00-865.69)
Septicemia	7	7.81# (3.14-16.10)	6	3.49# (1.28-7.60)	5	4.89# (1.59-11.42)	1	1.98 (0.05-11.03)	19	4.59# (2.76-7.16)
Other Infectious Diseases	7	13.43# (5.40-27.68)	6	6.05# (2.22-13.17)	1	1.81 (0.05-10.07)	1	4.04 (0.10-22.51)	15	6.48# (3.63-10.69)
Diabetes Mellitus	3	1.55 (0.32-4.54)	2	0.55 (0.07-1.97)	1	0.48 (0.01-2.65)	1	1.04 (0.03-5.79)	7	0.81 (0.32-1.67)
Alzheimer’s	3	1.59 (0.33-4.65)	2	0.53 (0.06-1.92)	6	2.30 (0.84-5.01)	2	1.18 (0.14-4.27)	13	1.31 (0.70-2.23)
Diseases of Heart	40	2.28# (1.63-3.10)	51	1.56# (1.16-2.05)	29	1.54# (1.03-2.21)	11	1.17 (0.58-2.09)	131	1.67# (1.40-1.98)

Hypertension without Heart Disease	2	3.19 (0.39-11.53)	0	0.00 (0.00-3.04)	1	1.26 (0.03-7.00)	0	0.00 (0.00-8.48)	3	0.98 (0.20-2.85)
Cerebrovascular Diseases	9	2.52# (1.15-4.78)	10	1.54 (0.74-2.83)	5	1.32 (0.43-3.09)	4	2.05 (0.56-5.24)	28	1.77# (1.18-2.56)
Atherosclerosis	0	0.00 (0.00-15.62)	0	0.00 (0.00-9.34)	0	0.00 (0.00-17.86)	0	0.00 (0.00-38.1)	0	0.00 (0.00-3.95)
Aortic Aneurysm and Dissection	0	0.00 (0.00-10.04)	1	1.54 (0.04-8.57)	0	0.00 (0.00-11.13)	1	7.13 (0.18-39.71)	2	1.34 (0.16-4.85)
Other Diseases of Arteries, Arterioles, Capillaries	0	0.00 (0.00-14.79)	0	0.00 (0.00-7.96)	0	0.00 (0.00-13.67)	1	7.44 (0.19-41.43)	1	0.90 (0.02-4.99)
Pneumonia and Influenza	2	1.29 (0.16-4.68)	5	1.76 (0.57-4.10)	6	3.54# (1.30-7.71)	0	0.00 (0.00-4.35)	13	1.88 (1.00-3.21)
Chronic Obstructive Pulmonary Disease	14	3.51# (1.92-5.89)	14	1.79 (0.98-3.01)	8	1.74 (0.75-3.44)	2	0.90 (0.11-3.23)	38	2.04# (1.44-2.80)
Stomach and Duodenal Ulcers	0	0.00 (0.00-41.17)	0	0.00 (0.00-22.92)	1	11.46 (0.29-63.86)	0	0.00 (0.00-90.66)	1	2.64 (0.07-14.72)
Chronic Liver	1	1.47	2	1.50	2	2.96	1	3.54	6	2.02

Disease and Cirrhosis		(0.04-8.17)		(0.18-5.42)		(0.36-10.69)		(0.09-19.73)		(0.74-4.39)
Nephritis, Nephrotic Syndrome and Nephrosis	5	3.99# (1.30-9.32)	2	0.83 (0.10-3.00)	1	0.67 (0.02-3.74)	0	0.00 (0.00-5.00)	8	1.36 (0.59-2.68)
Complications of Pregnancy, Childbirth, Puerperium	0	0.00 (0.00-10,196.17)	0	0.00 (0.00-5,814.34)	0	0.00 (0.00-19,826.21)	0	0.00 (0.00-115,008.42)	0	0.00 (0.00-3,037.69)
Congenital Anomalies	0	0.00 (0.00-67.43)	0	0.00 (0.00-35.95)	0	0.00 (0.00-70.82)	0	0.00 (0.00-159.17)	0	0.00 (0.00-15.86)
Certain Conditions Originating in Perinatal Period	0	0.00 (0.00-15,441.74)	0	0.00 (0.00-7,983.93)	0	0.00 (0.00-16,011.85)	0	0.00 (0.00-36,458.97)	0	0.00 (0.00-3,572.79)
Symptoms, Signs and Ill-Defined Conditions	4	6.28# (1.71-16.07)	2	1.66 (0.20-5.99)	1	1.30 (0.03-7.24)	0	0.00 (0.00-9.25)	7	2.32 (0.93-4.79)
Accidents and Adverse Effects	4	2.23 (0.61-5.72)	8	2.27 (0.98-4.48)	0	0.00 (0.00-1.82)	2	1.94 (0.24-7.02)	14	1.67 (0.91-2.81)
Suicide and Self-Inflicted Injury	1	2.00 (0.05-11.12)	1	1.01 (0.03-5.64)	0	0.00 (0.00-7.28)	0	0.00 (0.00-17.48)	2	0.91 (0.11-3.27)

Homicide and Legal Intervention	0	0.00 (0.00-49.08)	1	7.70 (0.19-42.89)	0	0.00 (0.00-64.02)	0	0.00 (0.00-161.97)	1	3.50 (0.09-19.52)
Other Cause of Death	44	5.03# (3.65-6.75)	34	1.95# (1.35-2.72)	17	1.52 (0.88-2.43)	5	0.81 (0.26-1.88)	100	2.29# (1.87-2.79)

1 number of cancer patients who died due to each cause of death.

2 95% Confidence interval.

# P value less than .05.

Supplementary table 14: Standardized mortality ratio (SMR) for each cause of death following esophageal cancer diagnosis in Grade II.

Cause of Death	Less than a year		1-5 years		5-10 years		More than 10 years		Total	
	Observed[1]	SMR (95% CI[2])	Observed[1]	SMR (95% CI[2])	Observed[1]	SMR (95% CI[2])	Observed[1]	SMR (95% CI[2])	Observed[1]	SMR (95% CI[2])
All Causes of Death	9,053	22.73# (22.26-23.20)	5,889	10.76# (10.49-11.04)	783	2.88# (2.68-3.09)	262	2.04# (1.80-2.31)	15,987	11.88# (11.70-12.07)
All Malignant Cancer Causes of Death	8,091	82.52# (80.73-84.34)	5,123	37.57# (36.55-38.61)	462	7.10# (6.47-7.78)	117	4.09# (3.38-4.90)	13,793	42.04# (41.34-42.75)

Esophageal Cancer Deaths	7,430	2,462.81# (2,407.12-2,519.45)	4,579	1,067.45# (1,036.75-1,098.82)	324	159.36# (142.48-177.69)	61	71.22# (54.48-91.48)	12,394	1,215.55# (1,194.24-1,237.14)
Non-cancer Causes of Death										
In situ, benign or unknown behavior neoplasm	14	5.47# (2.99-9.18)	18	4.99# (2.96-7.89)	8	4.27# (1.84-8.42)	0	0.00 (0.00-4.11)	40	4.48# (3.20-6.10)
Tuberculosis	0	0.00 (0.00-33.36)	0	0.00 (0.00-26.96)	1	16.55 (0.42-92.22)	0	0.00 (0.00-137.09)	1	2.99 (0.08-16.64)
Syphilis	0	0.00 (0.00-546.62)	0	0.00 (0.00-419.01)	0	0.00 (0.00-862.14)	0	0.00 (0.00-1,891.59)	0	0.00 (0.00-169.36)
Septicemia	41	7.14# (5.12-9.69)	32	4.04# (2.76-5.70)	13	3.27# (1.74-5.60)	4	2.12 (0.58-5.42)	90	4.61# (3.71-5.67)
Other Infectious Diseases	29	8.33# (5.58-11.97)	10	2.13# (1.02-3.92)	7	3.21# (1.29-6.62)	0	0.00 (0.00-3.96)	46	4.08# (2.98-5.44)
Diabetes Mellitus	21	1.69# (1.04-2.58)	21	1.23 (0.76-1.88)	8	0.97 (0.42-1.91)	1	0.26 (0.01-1.46)	51	1.23 (0.91-1.61)
Alzheimer’s	5	0.42# (0.14-0.98)	7	0.41# (0.16-0.84)	14	1.42 (0.77-2.37)	5	0.89 (0.29-2.08)	31	0.70# (0.47-0.99)

Diseases of Heart	334	3.03# (2.71-3.37)	278	1.89# (1.68-2.13)	105	1.47# (1.21-1.78)	48	1.44# (1.06-1.91)	765	2.11# (1.97-2.27)
Hypertension without Heart Disease	7	1.71 (0.69-3.52)	11	1.92 (0.96-3.43)	3	0.98 (0.20-2.86)	2	1.27 (0.15-4.58)	23	1.59# (1.01-2.38)
Cerebrovascular Diseases	52	2.29# (1.71-3.00)	26	0.87 (0.57-1.28)	15	1.04 (0.58-1.71)	7	0.99 (0.40-2.04)	100	1.35# (1.10-1.64)
Atherosclerosis	7	4.79# (1.92-9.86)	6	3.42# (1.26-7.45)	0	0.00 (0.00-4.91)	1	3.13 (0.08-17.45)	14	3.27# (1.79-5.48)
Aortic Aneurysm and Dissection	5	2.19 (0.71-5.12)	9	3.07# (1.40-5.83)	2	1.57 (0.19-5.68)	1	1.90 (0.05-10.59)	17	2.43# (1.41-3.88)
Other Diseases of Arteries, Arterioles, Capillaries	6	3.80# (1.40-8.28)	4	1.89 (0.51-4.83)	2	1.93 (0.23-6.97)	0	0.00 (0.00-7.59)	12	2.30# (1.19-4.02)
Pneumonia and Influenza	50	5.16# (3.83-6.80)	30	2.37# (1.60-3.38)	11	1.76 (0.88-3.15)	7	2.34 (0.94-4.83)	98	3.10# (2.52-3.78)
Chronic Obstructive Pulmonary Disease	80	3.26# (2.58-4.05)	91	2.61# (2.10-3.20)	32	1.81# (1.24-2.55)	25	3.01# (1.95-4.44)	228	2.67# (2.33-3.04)
Stomach and	1	1.77	2	2.73	1	2.99	0	0.00	4	2.24

Duodenal Ulcers		(0.04-9.86)		(0.33-9.87)		(0.08-16.68)		(0.00-24.27)		(0.61-5.74)
Chronic Liver Disease and Cirrhosis	25	5.77#<br>(3.73-8.52)	11	1.79<br>(0.89-3.20)	6	2.20<br>(0.81-4.78)	2	1.78<br>(0.22-6.42)	44	3.07#<br>(2.23-4.12)
Nephritis, Nephrotic Syndrome and Nephrosis	15	1.87#<br>(1.05-3.08)	13	1.18<br>(0.63-2.02)	3	0.53<br>(0.11-1.56)	5	1.84<br>(0.60-4.30)	36	1.32<br>(0.92-1.82)
Complications of Pregnancy, Childbirth, Puerperium	0	0.00<br>(0.00-1,842.66)	0	0.00<br>(0.00-1,439.22)	0	0.00<br>(0.00-4,117.80)	0	0.00<br>(0.00-19,650.16)	0	0.00<br>(0.00-653.06)
Congenital Anomalies	2	5.78<br>(0.70-20.86)	0	0.00<br>(0.00-7.81)	0	0.00<br>(0.00-17.77)	0	0.00<br>(0.00-42.14)	2	1.80<br>(0.22-6.49)
Certain Conditions Originating in Perinatal Period	0	0.00<br>(0.00-2,422.74)	0	0.00<br>(0.00-1,752.15)	0	0.00<br>(0.00-4,064.75)	0	0.00<br>(0.00-9,582.83)	0	0.00<br>(0.00-749.71)
Symptoms, Signs and Ill-Defined Conditions	30	7.40#<br>(4.99-10.56)	17	3.05#<br>(1.78-4.88)	3	1.04<br>(0.21-3.03)	4	3.06<br>(0.83-7.83)	54	3.90#<br>(2.93-5.09)
Accidents and Adverse Effects	27	2.39#<br>(1.57-3.47)	21	1.33<br>(0.82-2.03)	18	2.31#<br>(1.37-3.65)	3	0.81<br>(0.17-2.38)	69	1.79#<br>(1.39-2.26)

Suicide and Self-Inflicted Injury	22	7.12# (4.46-10.77)	7	1.60 (0.64-3.30)	1	0.50 (0.01-2.81)	4	4.92# (1.34-12.59)	34	3.31# (2.29-4.63)
Homicide and Legal Intervention	0	0.00 (0.00-7.00)	0	0.00 (0.00-5.81)	0	0.00 (0.00-15.42)	0	0.00 (0.00-40.98)	0	0.00 (0.00-2.47)
Other Cause of Death	189	3.43# (2.96-3.96)	152	1.93# (1.63-2.26)	68	1.59# (1.23-2.01)	26	1.19 (0.78-1.75)	435	2.19# (1.99-2.41)

1 number of cancer patients who died due to each cause of death.

2 95% Confidence interval.

# P value less than .05.

Supplementary table 15: Standardized mortality ratio (SMR) for each cause of death following esophageal cancer diagnosis in Grade III.

Cause of Death	Less than a year		1-5 years		5-10 years		More than 10 years		Total	
	Observed[1]	SMR (95% CI[2])	Observed[1]	SMR (95% CI[2])	Observed[1]	SMR (95% CI[2])	Observed[1]	SMR (95% CI[2])	Observed[1]	SMR (95% CI[2])
All Causes of Death	14,127	31.21# (30.70-31.73)	6,620	14.28# (13.93-14.62)	655	3.07# (2.84-3.31)	214	1.99# (1.73-2.27)	21,616	17.47# (17.24-17.70)

All Malignant Cancer Causes of Death	12,967	115.29# (113.31-117.29)	5,925	50.14# (48.88-51.44)	352	6.86# (6.16-7.62)	80	3.44# (2.73-4.28)	19,324	63.31# (62.42-64.21)
Esophageal Cancer Deaths	11,767	3,301.49# (3,242.11-3,361.6 9)	5,323	1,390.61# (1,353.50-1,42 8.48)	235	145.16# (127.19-164.9 5)	39	54.52# (38.77-74.53)	17,364	1,785.27# (1,758.82-1, 812.03)
Non-cancer Causes of Death										
In situ, benign or unknown behavior neoplasm	23	7.85# (4.97-11.77)	13	4.25# (2.26-7.26)	2	1.37 (0.17-4.95)	0	0.00 (0.00-4.96)	38	4.64# (3.28-6.36)
Tuberculosis	1	8.33 (0.21-46.44)	0	0.00 (0.00-32.31)	0	0.00 (0.00-78.32)	0	0.00 (0.00-184.55)	1	3.32 (0.08-18.50)
Syphilis	0	0.00 (0.00-511.58)	0	0.00 (0.00-506.7)	0	0.00 (0.00-1,113.4 5)	0	0.00 (0.00-2,595.6 9)	0	0.00 (0.00-191.88)
Septicemia	47	7.30# (5.36-9.70)	29	4.34# (2.90-6.23)	8	2.57# (1.11-5.07)	4	2.59 (0.71-6.64)	88	4.95# (3.97-6.10)
Other Infectious Diseases	22	5.73# (3.59-8.68)	15	3.77# (2.11-6.22)	2	1.16 (0.14-4.19)	0	0.00 (0.00-4.80)	39	3.78# (2.69-5.17)
Diabetes Mellitus	29	2.07#	12	0.82	4	0.62	2	0.65	47	1.23

		(1.38-2.97)		(0.42-1.43)		(0.17-1.58)		(0.08-2.36)		(0.90-1.64)
Alzheimer’s	8	0.61<br>(0.27-1.21)	9	0.67<br>(0.30-1.26)	8	1.04<br>(0.45-2.05)	7	1.40<br>(0.56-2.89)	32	0.82<br>(0.56-1.15)
Diseases of Heart	412	3.27#<br>(2.96-3.60)	233	1.88#<br>(1.64-2.13)	109	1.95#<br>(1.60-2.35)	49	1.73#<br>(1.28-2.28)	803	2.40#<br>(2.24-2.57)
Hypertension without Heart Disease	9	2.01<br>(0.92-3.81)	7	1.49<br>(0.60-3.07)	6	2.51<br>(0.92-5.47)	5	3.77#<br>(1.22-8.80)	27	2.09#<br>(1.38-3.05)
Cerebrovascular Diseases	62	2.43#<br>(1.86-3.12)	37	1.50#<br>(1.05-2.06)	17	1.49<br>(0.87-2.39)	6	1.00<br>(0.37-2.18)	122	1.80#<br>(1.50-2.15)
Atherosclerosis	5	2.96<br>(0.96-6.91)	0	0.00<br>(0.00-2.54)	2	3.34<br>(0.40-12.06)	0	0.00<br>(0.00-12.9)	7	1.74<br>(0.70-3.58)
Aortic Aneurysm and Dissection	7	2.63#<br>(1.06-5.42)	3	1.18<br>(0.24-3.45)	0	0.00<br>(0.00-3.67)	0	0.00<br>(0.00-8.43)	10	1.50<br>(0.72-2.77)
Other Diseases of Arteries, Arterioles, Capillaries	2	1.12<br>(0.14-4.04)	1	0.56<br>(0.01-3.12)	1	1.23<br>(0.03-6.86)	2	4.90<br>(0.59-17.69)	6	1.25<br>(0.46-2.72)
Pneumonia and Influenza	53	4.82#<br>(3.61-6.31)	34	3.22#<br>(2.23-4.50)	10	2.04<br>(0.98-3.75)	4	1.58<br>(0.43-4.04)	101	3.48#<br>(2.84-4.23)
Chronic	96	3.41#	86	2.89#	43	3.09#	14	2.03#	239	3.04#

Obstructive Pulmonary Disease		(2.76-4.16)		(2.31-3.57)		(2.24-4.17)		(1.11-3.40)		(2.66-3.45)
Stomach and Duodenal Ulcers	3	4.65 (0.96-13.58)	3	4.83 (1.00-14.12)	0	0.00 (0.00-13.96)	0	0.00 (0.00-28.81)	6	3.62# (1.33-7.87)
Chronic Liver Disease and Cirrhosis	27	5.35# (3.52-7.78)	7	1.28 (0.51-2.64)	4	1.81 (0.49-4.63)	1	1.05 (0.03-5.85)	39	2.85# (2.03-3.90)
Nephritis, Nephrotic Syndrome and Nephrosis	25	2.78# (1.80-4.11)	7	0.76 (0.30-1.56)	8	1.82 (0.78-3.58)	4	1.82 (0.50-4.65)	44	1.77# (1.29-2.38)
Complications of Pregnancy, Childbirth, Puerperium	1	588.25# (14.89-3,277.51)	0	0.00 (0.00-2,524.23)	0	0.00 (0.00-9,525.36)	0	0.00 (0.00-21,979.49)	1	269.07# (6.81-1,499.18)
Congenital Anomalies	0	0.00 (0.00-9.31)	1	2.44 (0.06-13.58)	1	6.03 (0.15-33.62)	0	0.00 (0.00-49.98)	2	1.91 (0.23-6.91)
Certain Conditions Originating in Perinatal Period	0	0.00 (0.00-2,090.44)	0	0.00 (0.00-1,977.57)	0	0.00 (0.00-4,851.20)	0	0.00 (0.00-11,700.53)	0	0.00 (0.00-783.92)
Symptoms, Signs and Ill-Defined	22	4.85#	15	3.29#	2	0.88	3	2.52	42	3.35#

Conditions		(3.04-7.34)		(1.84-5.43)		(0.11-3.18)		(0.52-7.36)		(2.41-4.52)
Accidents and Adverse Effects	22	1.71# (1.07-2.59)	23	1.70# (1.08-2.55)	10	1.63 (0.78-3.00)	4	1.28 (0.35-3.27)	59	1.65# (1.26-2.13)
Suicide and Self-Inflicted Injury	42	11.35# (8.18-15.35)	16	4.05# (2.31-6.57)	5	3.12# (1.01-7.29)	0	0.00 (0.00-5.35)	63	6.34# (4.87-8.11)
Homicide and Legal Intervention	0	0.00 (0.00-6.50)	2	3.70 (0.45-13.38)	0	0.00 (0.00-19.85)	0	0.00 (0.00-53.12)	2	1.47 (0.18-5.30)
Other Cause of Death	242	3.93# (3.45-4.45)	142	2.17# (1.83-2.56)	61	1.83# (1.40-2.35)	29	1.56# (1.04-2.24)	474	2.65# (2.41-2.90)

1 number of cancer patients who died due to each cause of death.

2 95% Confidence interval.

# P value less than .05.

Supplementary table 16: Standardized mortality ratio (SMR) for each cause of death following esophageal cancer diagnosis in Grade IV.

Cause of Death	Less than a year		1-5 years		5-10 years		More than 10 years		Total	
	Observed[1]	SMR (95% CI[2])	Observed[1]	SMR (95% CI[2])	Observed[1]	SMR (95% CI[2])	Observed[1]	SMR (95% CI[2])	Observed[1]	SMR (95% CI[2])

All Causes of Death	560	30.05# (27.61-32.65)	242	10.41# (9.14-11.81)	33	2.36# (1.63-3.32)	15	2.46# (1.38-4.06)	850	13.72# (12.82-14.68)
All Malignant Cancer Causes of Death	512	114.01# (104.34-124.32)	219	39.31# (34.27-44.87)	16	5.17# (2.96-8.40)	1	0.75 (0.02-4.19)	748	51.63# (48.00-55.47)
Esophageal Cancer Deaths	447	3,322.00# (3,021.14-3,644.72)	193	1,145.31# (989.42-1,318.78)	13	139.35# (74.20-238.29)	1	23.94 (0.61-133.37)	654	1,492.67# (1,380.45-1,611.59)
Non-cancer Causes of Death										
In situ, benign or unknown behavior neoplasm	4	33.33# (9.08-85.34)	1	6.41 (0.16-35.74)	2	21.26# (2.57-76.79)	0	0.00 (0.00-83.80)	7	16.91# (6.80-34.84)
Tuberculosis	0	0.00 (0.00-755.17)	0	0.00 (0.00-644.98)	0	0.00 (0.00-1,314.01)	0	0.00 (0.00-3,821.59)	0	0.00 (0.00-256.59)
Syphilis	0	0.00 (0.00-13,781.65)	0	0.00 (0.00-13,535.11)	0	0.00 (0.00-24,545.61)	0	0.00 (0.00-46,667.48)	0	0.00 (0.00-4,793.61)
Septicemia	2	7.64	1	3.06	0	0.00	0	0.00	3	3.43

		(0.92-27.59)		(0.08-17.05)		(0.00-18.66)		(0.00-41.65)		(0.71-10.02)
Other Infectious Diseases	1	6.74<br>(0.17-37.55)	0	0.00<br>(0.00-19.71)	0	0.00<br>(0.00-34.67)	0	0.00<br>(0.00-88.44)	1	2.07<br>(0.05-11.52)
Diabetes Mellitus	1	1.77<br>(0.04-9.84)	0	0.00<br>(0.00-5.30)	0	0.00<br>(0.00-9.10)	0	0.00<br>(0.00-21.25)	1	0.54<br>(0.01-3.03)
Alzheimer's	0	0.00<br>(0.00-6.29)	0	0.00<br>(0.00-4.68)	1	1.62<br>(0.04-9.04)	1	3.77<br>(0.10-21.01)	2	0.89<br>(0.11-3.20)
Diseases of Heart	18	3.43#<br>(2.03-5.42)	11	1.73<br>(0.86-3.10)	7	1.91<br>(0.77-3.93)	6	3.66#<br>(1.34-7.96)	42	2.48#<br>(1.79-3.36)
Hypertension without Heart Disease	1	5.36<br>(0.14-29.88)	0	0.00<br>(0.00-15.28)	0	0.00<br>(0.00-21.81)	0	0.00<br>(0.00-51.93)	1	1.50<br>(0.04-8.34)
Cerebrovascular Diseases	4	3.61<br>(0.98-9.24)	1	0.77<br>(0.02-4.27)	1	1.24<br>(0.03-6.92)	1	3.05<br>(0.08-16.98)	7	1.97<br>(0.79-4.07)
Atherosclerosis	2	26.22#<br>(3.18-94.73)	0	0.00<br>(0.00-45.26)	0	0.00<br>(0.00-86.14)	0	0.00<br>(0.00-232.62)	2	9.24#<br>(1.12-33.38)
Aortic Aneurysm and Dissection	1	8.95<br>(0.23-49.85)	0	0.00<br>(0.00-29.18)	0	0.00<br>(0.00-58.39)	0	0.00<br>(0.00-147.57)	1	3.06<br>(0.08-17.07)
Other Diseases of Arteries, Arterioles,	0	0.00<br>(0.00-48.69)	0	0.00<br>(0.00-41.00)	0	0.00<br>(0.00-68.88)	0	0.00<br>(0.00-159.59)	0	0.00<br>(0.00-15.22)

Capillaries										
Pneumonia and Influenza	0	0.00 (0.00-7.92)	0	0.00 (0.00-6.48)	2	6.10 (0.74-22.02)	0	0.00 (0.00-25.75)	2	1.33 (0.16-4.80)
Chronic Obstructive Pulmonary Disease	1	0.86 (0.02-4.81)	1	0.67 (0.02-3.75)	0	0.00 (0.00-4.13)	1	2.55 (0.06-14.19)	3	0.76 (0.16-2.23)
Stomach and Duodenal Ulcers	0	0.00 (0.00-135.54)	0	0.00 (0.00-117.16)	0	0.00 (0.00-211.18)	0	0.00 (0.00-524.28)	0	0.00 (0.00-44.33)
Chronic Liver Disease and Cirrhosis	0	0.00 (0.00-19.12)	0	0.00 (0.00-15.75)	0	0.00 (0.00-29.02)	1	20.66 (0.52-115.11)	1	1.66 (0.04-9.25)
Nephritis, Nephrotic Syndrome and Nephrosis	0	0.00 (0.00-10.18)	0	0.00 (0.00-7.90)	1	3.60 (0.09-20.04)	0	0.00 (0.00-28.32)	1	0.81 (0.02-4.50)
Complications of Pregnancy, Childbirth, Puerperium	0	0.00 (0.00-47,906.75)	0	0.00 (0.00-66,869.94)	0	0.00 (0.00-231,694.18)	0	0.00 (0.00-5,900,866.63)	0	0.00 (0.00-24,805.41)
Congenital Anomalies	0	0.00 (0.00-234.14)	0	0.00 (0.00-198.96)	0	0.00 (0.00-368.52)	0	0.00 (0.00-966.25)	0	0.00 (0.00-76.65)

Certain Conditions Originating in Perinatal Period	0	0.00 (0.00-57,687.28)	0	0.00 (0.00-49,172.59)	0	0.00 (0.00-88,505.47)	0	0.00 (0.00-219,845.10)	0	0.00 (0.00-18,685.03)
Symptoms, Signs and Ill-Defined Conditions	0	0.00 (0.00-19.08)	1	4.01 (0.10-22.35)	0	0.00 (0.00-21.72)	0	0.00 (0.00-57.61)	1	1.48 (0.04-8.24)
Accidents and Adverse Effects	0	0.00 (0.00-7.21)	1	1.54 (0.04-8.59)	0	0.00 (0.00-9.47)	1	5.76 (0.15-32.07)	2	1.16 (0.14-4.19)
Suicide and Self-Inflicted Injury	2	14.09# (1.71-50.89)	1	5.91 (0.15-32.91)	0	0.00 (0.00-41.86)	0	0.00 (0.00-98.72)	3	6.87# (1.42-20.07)
Homicide and Legal Intervention	0	0.00 (0.00-174.85)	0	0.00 (0.00-168.38)	0	0.00 (0.00-379.68)	0	0.00 (0.00-1,131.76)	0	0.00 (0.00-65.90)
Other Cause of Death	11	4.30# (2.15-7.69)	5	1.47 (0.48-3.42)	3	1.29 (0.27-3.76)	3	2.87 (0.59-8.39)	22	2.35# (1.48-3.56)

1 number of cancer patients who died due to each cause of death.

2 95% Confidence interval.

# P value less than .05.

Supplementary table 17: Standardized mortality ratio (SMR) for each cause of death following esophageal cancer diagnosis in patients who underwent cancer-directed surgery.

Cause of Death	Less than a year		1-5 years		5-10 years		More than 10 years		Total	
	Observed[1]	SMR (95% CI[2])	Observed[1]	SMR (95% CI[2])	Observed[1]	SMR (95% CI[2])	Observed[1]	SMR (95% CI[2])	Observed[1]	SMR (95% CI[2])
All Causes of Death	3,145	10.64# (10.27-11.02)	5,273	7.74# (7.53-7.95)	1,137	2.48# (2.34-2.63)	440	1.75# (1.59-1.92)	9,995	5.93# (5.81-6.05)
All Malignant Cancer Causes of Death	2,577	30.87# (29.69-32.09)	4,503	24.28# (23.58-25.00)	649	5.68# (5.25-6.13)	180	3.24# (2.78-3.74)	7,909	18.02# (17.63-18.43)
Esophageal Cancer Deaths	2,359	802.88# (770.80-835.94)	3,992	619.01# (599.95-638.51)	448	117.29# (106.68-128.67)	99	56.55# (45.96-68.84)	6,898	461.17# (450.35-472.19)
Non-cancer Causes of Death										
In situ, benign or unknown behavior neoplasm	2	1.06 (0.13-3.84)	14	3.10# (1.69-5.20)	10	3.13# (1.50-5.75)	1	0.57 (0.01-3.15)	27	2.38# (1.57-3.46)
Tuberculosis	0	0.00 (0.00-47.65)	0	0.00 (0.00-23.01)	1	10.49 (0.27-58.46)	0	0.00 (0.00-81.22)	1	2.64 (0.07-14.72)
Syphilis	0	0.00 (0.00-843.68)	0	0.00 (0.00-373.22)	0	0.00 (0.00-582.68)	0	0.00 (0.00-1,125.2	0	0.00 (0.00-154.57

								2)		)
Septicemia	45	10.88# (7.94-14.56)	38	3.93# (2.78-5.39)	17	2.59# (1.51-4.15)	4	1.11 (0.30-2.83)	104	4.34# (3.54-5.25)
Other Infectious Diseases	9	3.07# (1.40-5.83)	10	1.57 (0.75-2.89)	4	1.06 (0.29-2.71)	1	0.56 (0.01-3.09)	24	1.61# (1.03-2.40)
Diabetes Mellitus	5	0.51 (0.17-1.19)	14	0.64 (0.35-1.07)	7	0.50 (0.20-1.03)	4	0.55 (0.15-1.42)	30	0.57# (0.38-0.81)
Alzheimer's	0	0.00# (0.00-0.63)	8	0.48# (0.21-0.95)	16	1.07 (0.61-1.74)	17	1.53 (0.89-2.45)	41	0.84 (0.61-1.15)
Diseases of Heart	159	2.04# (1.73-2.38)	251	1.42# (1.25-1.60)	165	1.39# (1.19-1.62)	94	1.42# (1.15-1.74)	669	1.52# (1.41-1.64)
Hypertension without Heart Disease	5	1.96 (0.64-4.58)	7	1.10 (0.44-2.27)	7	1.47 (0.59-3.02)	5	1.68 (0.54-3.91)	24	1.44 (0.92-2.14)
Cerebrovascular Diseases	29	2.01# (1.35-2.89)	31	0.93 (0.63-1.32)	30	1.30 (0.87-1.85)	13	0.96 (0.51-1.63)	103	1.22 (0.99-1.48)
Atherosclerosis	1	1.19 (0.03-6.60)	1	0.55 (0.01-3.04)	1	0.84 (0.02-4.67)	1	1.57 (0.04-8.75)	4	0.89 (0.24-2.27)
Aortic Aneurysm and Dissection	3	1.65 (0.34-4.82)	5	1.31 (0.43-3.06)	0	0.00 (0.00-1.67)	2	1.93 (0.23-6.97)	10	1.13 (0.54-2.07)

Other Diseases of Arteries, Arterioles, Capillaries	1	0.91 (0.02-5.09)	5	1.98 (0.64-4.63)	3	1.77 (0.36-5.16)	2	2.12 (0.26-7.67)	11	1.76 (0.88-3.15)
Pneumonia and Influenza	34	5.74# (3.98-8.02)	30	2.14# (1.44-3.05)	20	1.98# (1.21-3.06)	10	1.72 (0.82-3.16)	94	2.62# (2.12-3.21)
Chronic Obstructive Pulmonary Disease	47	2.47# (1.82-3.29)	89	1.98# (1.59-2.44)	47	1.53# (1.13-2.04)	29	1.77# (1.19-2.54)	212	1.91# (1.66-2.19)
Stomach and Duodenal Ulcers	0	0.00 (0.00-8.91)	1	1.11 (0.03-6.19)	2	3.54 (0.43-12.79)	0	0.00 (0.00-12.40)	3	1.38 (0.28-4.03)
Chronic Liver Disease and Cirrhosis	16	3.45# (1.97-5.60)	10	1.03 (0.49-1.90)	6	1.15 (0.42-2.50)	3	1.31 (0.27-3.83)	35	1.60# (1.11-2.23)
Nephritis, Nephrotic Syndrome and Nephrosis	13	2.44# (1.30-4.17)	18	1.40 (0.83-2.21)	9	0.98 (0.45-1.87)	7	1.36 (0.55-2.80)	47	1.45# (1.06-1.92)
Complications of Pregnancy, Childbirth, Puerperium	0	0.00 (0.00-2,016.67)	0	0.00 (0.00-1,205.13)	0	0.00 (0.00-3,612.04)	0	0.00 (0.00-12,797.70)	0	0.00 (0.00-595.01)
Congenital	0	0.00	0	0.00	1	2.65	0	0.00	1	0.64

Anomalies		(0.00-11.11)		(0.00-5.36)		(0.07-14.79)		(0.00-21.16)		(0.02-3.55)
Certain Conditions Originating in Perinatal Period	0	0.00 (0.00-2,352.01)	0	0.00 (0.00-1,140.92)	0	0.00 (0.00-2,139.73)	0	0.00 (0.00-4,903.11)	0	0.00 (0.00-506.85)
Symptoms, Signs and Ill-Defined Conditions	18	7.00# (4.15-11.06)	14	2.23# (1.22-3.74)	2	0.43 (0.05-1.56)	6	2.29 (0.84-4.98)	40	2.48# (1.77-3.38)
Accidents and Adverse Effects	9	0.95 (0.43-1.80)	36	1.69# (1.18-2.34)	24	1.76# (1.13-2.62)	8	1.08 (0.47-2.13)	77	1.48# (1.17-1.86)
Suicide and Self-Inflicted Injury	11	3.24# (1.62-5.80)	12	1.69 (0.88-2.96)	6	1.55 (0.57-3.37)	1	0.59 (0.01-3.28)	30	1.87# (1.26-2.67)
Homicide and Legal Intervention	0	0.00 (0.00-7.35)	1	1.07 (0.03-5.96)	0	0.00 (0.00-8.63)	0	0.00 (0.00-22.17)	1	0.49 (0.01-2.74)
Other Cause of Death	161	4.34# (3.70-5.07)	175	1.88# (1.61-2.18)	110	1.56# (1.29-1.89)	52	1.21 (0.90-1.59)	498	2.05# (1.87-2.24)

1 number of cancer patients who died due to each cause of death.

2 95% Confidence interval.

# P value less than .05.

Supplementary table 18: Standardized mortality ratio (SMR) for each cause of death following esophageal cancer diagnosis in patients whose surgery was not recommended.

Cause of Death	Less than a year		1-5 years		5-10 years		More than 10 years		Total	
	Observed[1]	SMR (95% CI[2])	Observed[1]	SMR (95% CI[2])	Observed[1]	SMR (95% CI[2])	Observed[1]	SMR (95% CI[2])	Observed[1]	SMR (95% CI[2])
All Causes of Death	24,082	32.89# (32.47-33.31)	9,018	14.15# (13.86-14.45)	727	3.30# (3.07-3.55)	196	2.76# (2.38-3.17)	34,023	20.49# (20.27-20.71)
All Malignant Cancer Causes of Death	22,023	128.97# (127.27-130.68)	7,986	54.95# (53.75-56.17)	379	8.00# (7.22-8.85)	83	5.86# (4.67-7.27)	30,471	80.69# (79.79-81.60)
Esophageal Cancer Deaths	19,894	3,924.46# (3,870.11-3,979.3 8)	7,126	1,716.24# (1,676.62-1,75 6.56)	258	199.13# (175.57-224.9 7)	43	114.63# (82.96-154.40)	27,321	2,508.33# (2,478.67-2, 538.25)
Non-cancer Causes of Death										
In situ, benign or unknown behavior neoplasm	63	13.22# (10.16-16.91)	26	6.14# (4.01-9.00)	3	2.02 (0.42-5.91)	0	0.00 (0.00-7.56)	92	8.39# (6.76-10.29)
Tuberculosis	1	5.12 (0.13-28.55)	0	0.00 (0.00-23.19)	0	0.00 (0.00-69.94)	0	0.00 (0.00-235.72)	1	2.37 (0.06-13.18)

Syphilis	0	0.00 (0.00-301.24)	0	0.00 (0.00-365.67)	0	0.00 (0.00-995.95)	0	0.00 (0.00-3,589.48)	0	0.00 (0.00-136.30)
Septicemia	65	6.15# (4.74-7.83)	40	4.31# (3.08-5.87)	9	2.77# (1.27-5.26)	3	2.93 (0.60-8.55)	117	4.85# (4.01-5.81)
Other Infectious Diseases	58	9.72# (7.38-12.56)	21	4.23# (2.62-6.47)	6	3.61# (1.33-7.87)	0	0.00 (0.00-7.48)	85	6.49# (5.19-8.03)
Diabetes Mellitus	51	2.30# (1.71-3.02)	29	1.52# (1.02-2.19)	6	0.93 (0.34-2.03)	3	1.52 (0.31-4.45)	89	1.79# (1.44-2.21)
Alzheimer's	17	0.69 (0.40-1.10)	15	0.63 (0.35-1.04)	11	1.13 (0.56-2.02)	2	0.55 (0.07-1.98)	45	0.73# (0.53-0.97)
Diseases of Heart	720	3.51# (3.26-3.78)	366	2.08# (1.87-2.31)	132	2.23# (1.87-2.64)	38	1.99# (1.41-2.73)	1,256	2.74# (2.59-2.89)
Hypertension without Heart Disease	30	3.78# (2.55-5.40)	14	1.94# (1.06-3.26)	5	1.80 (0.58-4.19)	1	1.06 (0.03-5.88)	50	2.65# (1.97-3.49)
Cerebrovascular Diseases	115	2.67# (2.20-3.20)	59	1.58# (1.20-2.04)	18	1.40 (0.83-2.21)	7	1.67 (0.67-3.45)	199	2.04# (1.77-2.35)
Atherosclerosis	13	4.63# (2.47-7.92)	7	3.03# (1.22-6.24)	1	1.45 (0.04-8.05)	0	0.00 (0.00-18.12)	21	3.49# (2.16-5.34)

Aortic Aneurysm and Dissection	13	3.24# (1.72-5.53)	5	1.52 (0.50-3.56)	2	2.06 (0.25-7.45)	0	0.00 (0.00-13.33)	20	2.34# (1.43-3.62)
Other Diseases of Arteries, Arterioles, Capillaries	9	3.06# (1.40-5.81)	5	1.97 (0.64-4.59)	1	1.15 (0.03-6.41)	1	3.66 (0.09-20.37)	16	2.42# (1.38-3.92)
Pneumonia and Influenza	71	3.80# (2.97-4.80)	36	2.21# (1.55-3.06)	9	1.62 (0.74-3.08)	6	3.32# (1.22-7.23)	122	2.89# (2.40-3.44)
Chronic Obstructive Pulmonary Disease	180	4.01# (3.45-4.64)	115	2.89# (2.39-3.47)	46	3.36# (2.46-4.48)	18	4.13# (2.45-6.52)	359	3.50# (3.14-3.88)
Stomach and Duodenal Ulcers	5	4.88# (1.58-11.38)	4	4.68# (1.28-11.99)	0	0.00 (0.00-13.57)	1	11.90 (0.30-66.29)	10	4.47# (2.14-8.23)
Chronic Liver Disease and Cirrhosis	55	7.89# (5.94-10.27)	17	3.09# (1.80-4.94)	7	4.15# (1.67-8.56)	3	6.16# (1.27-17.99)	82	5.60# (4.45-6.95)
Nephritis, Nephrotic Syndrome and Nephrosis	42	2.78# (2.00-3.75)	17	1.26 (0.73-2.02)	7	1.46 (0.59-3.01)	3	1.95 (0.40-5.70)	69	1.97# (1.54-2.50)
Complications of Pregnancy,	2	617.61# (74.80-2,231.02)	0	0.00 (0.00-1,301.48)	0	0.00 (0.00-4,215.1	0	0.00 (0.00-17,064.	2	279.17# (33.81-1,008

Childbirth, Puerperium						2)		40)		.47)
Congenital Anomalies	3	5.20# (1.07-15.19)	1	2.16 (0.05-12.01)	0	0.00 (0.00-25.58)	0	0.00 (0.00-85.11)	4	3.26 (0.89-8.33)
Certain Conditions Originating in Perinatal Period	0	0.00 (0.00-1,489.62)	1	511.88# (12.96-2,852.01)	0	0.00 (0.00-6,200.72)	0	0.00(0.00-19,571.68)	1	191.81# (4.86-1,068.72)
Symptoms, Signs and Ill-Defined Conditions	50	6.39# (4.74-8.42)	28	4.00# (2.66-5.77)	4	1.54 (0.42-3.94)	1	1.19 (0.03-6.65)	83	4.54# (3.62-5.63)
Accidents and Adverse Effects	43	2.12# (1.53-2.85)	24	1.39 (0.89-2.06)	10	1.67 (0.80-3.07)	3	1.49 (0.31-4.35)	80	1.75# (1.39-2.18)
Suicide and Self-Inflicted Injury	62	12.32# (9.45-15.80)	12	3.07# (1.58-5.35)	2	1.69 (0.21-6.12)	3	8.60# (1.77-25.13)	79	7.54# (5.97-9.40)
Homicide and Legal Intervention	0	0.00 (0.00-4.45)	5	8.55# (2.78-19.95)	0	0.00 (0.00-23.06)	0	0.00 (0.00-85.89)	5	3.09# (1.00-7.21)
Other Cause of Death	391	3.69# (3.34-4.08)	185	1.91# (1.65-2.21)	69	1.88# (1.46-2.38)	20	1.57 (0.96-2.42)	665	2.64# (2.44-2.85)

1 number of cancer patients who died due to each cause of death.

2 95% Confidence interval.

# P value less than .05.

Supplementary table 19: Standardized mortality ratio (SMR) for each cause of death following esophageal cancer diagnosis in patients who received radiotherapy.

Cause of Death	Less than a year		1-5 years		5-10 years		More than 10 years		Total	
	Observed[1]	SMR (95% CI[2])	Observed[1]	SMR (95% CI[2])	Observed[1]	SMR (95% CI[2])	Observed[1]	SMR (95% CI[2])	Observed[1]	SMR (95% CI[2])
All Causes of Death	14,168	19.94# (19.61-20.27)	10,984	12.66# (12.42-12.90)	1,252	3.44# (3.25-3.63)	387	2.52# (2.28-2.79)	26,791	12.78# (12.63-12.94)
All Malignant Cancer Causes of Death	12,829	72.31# (71.07-73.58)	9,658	44.71# (43.82-45.61)	708	8.12# (7.53-8.74)	163	4.69# (4.00-5.47)	23,358	45.32# (44.74-45.91)
Esophageal Cancer Deaths	11,843	2,126.00# (2,087.88-2,164.64)	8,759	1,290.99# (1,264.10-1,318.32)	487	180.83# (165.12-197.63)	85	79.68# (63.65-98.53)	21,174	1,313.92# (1,296.28-1,331.74)
Non-cancer Causes of Death										
In situ, benign or unknown behavior neoplasm	23	5.02# (3.18-7.54)	32	5.65# (3.86-7.97)	8	3.27# (1.41-6.45)	0	0.00 (0.00-3.49)	63	4.58# (3.52-5.86)

Tuberculosis	0	0.00 (0.00-19.13)	0	0.00 (0.00-17.05)	1	12.22 (0.31-68.06)	0	0.00 (0.00-119.23)	1	1.92 (0.05-10.67)
Syphilis	0	0.00 (0.00-303.43)	0	0.00 (0.00-251.90)	0	0.00 (0.00-569.66)	0	0.00 (0.00-1,557.94)	0	0.00 (0.00-103.49)
Septicemia	67	6.52# (5.05-8.27)	59	4.64# (3.53-5.99)	19	3.51# (2.12-5.49)	11	4.87# (2.43-8.71)	156	5.09# (4.32-5.95)
Other Infectious Diseases	34	5.43# (3.76-7.58)	21	2.80# (1.73-4.27)	8	2.65# (1.15-5.23)	0	0.00 (0.00-3.19)	63	3.51# (2.70-4.49)
Diabetes Mellitus	28	1.25 (0.83-1.81)	26	0.95 (0.62-1.39)	10	0.89 (0.43-1.63)	3	0.65 (0.13-1.91)	67	1.02 (0.79-1.30)
Alzheimer’s	3	0.15# (0.03-0.43)	14	0.52# (0.28-0.87)	17	1.29 (0.75-2.06)	5	0.77 (0.25-1.79)	39	0.58# (0.41-0.79)
Diseases of Heart	472	2.42# (2.20-2.64)	462	1.98# (1.81-2.17)	190	1.99# (1.72-2.29)	79	1.99# (1.57-2.47)	1,203	2.13# (2.02-2.26)
Hypertension without Heart Disease	12	1.66 (0.86-2.89)	15	1.63 (0.91-2.69)	7	1.65 (0.67-3.41)	4	2.13 (0.58-5.46)	38	1.68# (1.19-2.31)
Cerebrovascular Diseases	47	1.18 (0.87-1.57)	58	1.22 (0.93-1.58)	30	1.53# (1.03-2.18)	10	1.20 (0.57-2.20)	145	1.26# (1.06-1.48)
Atherosclerosis	7	2.81#	5	1.80	1	0.98	0	0.00	13	1.95#

		(1.13-5.79)		(0.59-4.21)		(0.02-5.45)		(0.00-9.64)		(1.04-3.33)
Aortic Aneurysm and Dissection	10	2.48# (1.19-4.56)	11	2.40# (1.20-4.29)	2	1.19 (0.14-4.29)	0	0.00 (0.00-5.84)	23	2.10# (1.33-3.15)
Other Diseases of Arteries, Arterioles, Capillaries	6	2.15 (0.79-4.68)	5	1.48 (0.48-3.46)	3	2.13 (0.44-6.23)	1	1.72 (0.04-9.57)	15	1.84# (1.03-3.03)
Pneumonia and Influenza	64	3.80# (2.93-4.85)	59	2.94# (2.24-3.79)	17	2.03# (1.19-3.26)	9	2.57# (1.17-4.87)	149	3.05# (2.58-3.59)
Chronic Obstructive Pulmonary Disease	103	2.34# (1.91-2.83)	147	2.68# (2.27-3.15)	75	3.22# (2.53-4.04)	35	3.54# (2.46-4.92)	360	2.73# (2.45-3.02)
Stomach and Duodenal Ulcers	2	2.01 (0.24-7.24)	4	3.45 (0.94-8.85)	1	2.23 (0.06-12.40)	0	0.00 (0.00-20.17)	7	2.51# (1.01-5.17)
Chronic Liver Disease and Cirrhosis	34	4.20# (2.91-5.87)	25	2.54# (1.64-3.75)	9	2.39# (1.09-4.53)	4	2.71 (0.74-6.95)	72	3.11# (2.43-3.91)
Nephritis, Nephrotic Syndrome and Nephrosis	28	1.96# (1.31-2.84)	19	1.08 (0.65-1.68)	10	1.31 (0.63-2.41)	6	1.88 (0.69-4.09)	63	1.48# (1.13-1.89)
Complications of Pregnancy,	0	0.00	0	0.00	0	0.00	0	0.00	0	0.00

Childbirth, Puerperium		(0.00-1,023.80)		(0.00-875.86)		(0.00-2,887.82)		(0.00-8,876.27)		(0.00-387.98)
Congenital Anomalies	0	0.00 (0.00-5.84)	1	1.32 (0.03-7.37)	1	3.48 (0.09-19.41)	0	0.00 (0.00-32.93)	2	1.12 (0.14-4.04)
Certain Conditions Originating in Perinatal Period	0	0.00 (0.00-1,302.42)	1	291.56# (7.38-1,624.45)	0	0.00 (0.00-2,862.34)	0	0.00 (0.00-7,396.40)	1	124.23# (3.15-692.16)
Symptoms, Signs and Ill-Defined Conditions	40	5.68# (4.06-7.73)	29	3.29# (2.21-4.73)	6	1.53 (0.56-3.32)	5	3.12# (1.01-7.28)	80	3.74# (2.97-4.66)
Accidents and Adverse Effects	24	1.17 (0.75-1.74)	42	1.67# (1.20-2.25)	19	1.82# (1.09-2.84)	7	1.56 (0.63-3.21)	92	1.52# (1.22-1.86)
Suicide and Self-Inflicted Injury	42	7.27# (5.24-9.83)	20	2.87# (1.75-4.43)	3	1.13 (0.23-3.29)	2	1.92 (0.23-6.93)	67	4.07# (3.16-5.17)
Homicide and Legal Intervention	0	0.00 (0.00-3.85)	5	4.81# (1.56-11.24)	0	0.00 (0.00-10.59)	0	0.00 (0.00-31.27)	5	2.03 (0.66-4.74)
Other Cause of Death	293	2.99# (2.66-3.35)	266	2.13# (1.88-2.40)	107	1.87# (1.54-2.27)	43	1.66# (1.20-2.24)	709	2.32# (2.15-2.50)

1 number of cancer patients who died due to each cause of death.

2 95% Confidence interval.

# P value less than .05.

Supplementary table 20: Standardized mortality ratio (SMR) for each cause of death following esophageal cancer diagnosis in patients who received chemotherapy.

Cause of Death	Less than a year		1-5 years		5-10 years		More than 10 years		Total	
	Observed[1]	SMR (95% CI[2])	Observed[1]	SMR (95% CI[2])	Observed[1]	SMR (95% CI[2])	Observed[1]	SMR (95% CI[2])	Observed[1]	SMR (95% CI[2])
All Causes of Death	15,133	21.96# (21.61-22.31)	12,636	14.30# (14.06-14.56)	1,331	3.44# (3.26-3.63)	399	2.45# (2.21-2.70)	29,499	13.90# (13.74-14.06)
All Malignant Cancer Causes of Death	13,814	75.26# (74.01-76.52)	11,299	49.65# (48.73-50.57)	780	8.30# (7.72-8.90)	172	4.64# (3.97-5.38)	26,065	48.07# (47.49-48.65)
Esophageal Cancer Deaths	12,667	2,087.68# (2,051.48-2,124.35)	10,136	1,379.48# (1,352.75-1,406.60)	537	181.88# (166.82-197.94)	89	78.14# (62.76-96.16)	23,429	1,338.30# (1,321.21-1,355.54)
Non-cancer Causes of Death										
In situ, benign or unknown behavior neoplasm	24	5.43# (3.48-8.07)	33	5.72# (3.93-8.03)	7	2.68# (1.08-5.51)	0	0.00 (0.00-3.27)	64	4.59# (3.54-5.86)

Tuberculosis	0	0.00 (0.00-19.37)	0	0.00 (0.00-16.61)	1	11.65 (0.30-64.93)	0	0.00 (0.00-113.21)	1	1.88 (0.05-10.49)
Syphilis	0	0.00 (0.00-307.51)	0	0.00 (0.00-245.80)	0	0.00 (0.00-553.77)	0	0.00 (0.00-1,470.95)	0	0.00 (0.00-101.98)
Septicemia	71	7.11# (5.55-8.97)	64	4.94# (3.81-6.31)	19	3.32# (2.00-5.18)	10	4.16# (2.00-7.65)	164	5.28# (4.50-6.15)
Other Infectious Diseases	42	6.40# (4.61-8.66)	23	2.91# (1.85-4.37)	9	2.80# (1.28-5.31)	0	0.00 (0.00-3.01)	74	3.92# (3.08-4.92)
Diabetes Mellitus	25	1.11 (0.72-1.63)	27	0.95 (0.63-1.39)	11	0.92 (0.46-1.64)	3	0.61 (0.13-1.79)	66	0.97 (0.75-1.24)
Alzheimer's	2	0.12# (0.01-0.43)	13	0.52# (0.27-0.88)	17	1.26 (0.73-2.01)	6	0.88 (0.32-1.91)	38	0.61# (0.43-0.84)
Diseases of Heart	431	2.34# (2.12-2.57)	455	1.94# (1.77-2.13)	200	1.98# (1.72-2.28)	81	1.92# (1.52-2.38)	1,167	2.08# (1.96-2.20)
Hypertension without Heart Disease	7	1.05 (0.42-2.17)	11	1.21 (0.60-2.17)	8	1.83 (0.79-3.60)	3	1.52 (0.31-4.44)	29	1.31 (0.88-1.88)
Cerebrovascular Diseases	53	1.47# (1.10-1.92)	59	1.26 (0.96-1.63)	26	1.27 (0.83-1.86)	10	1.13 (0.54-2.08)	148	1.32# (1.12-1.55)
Atherosclerosis	7	3.34#	4	1.52	1	0.94	0	0.00	12	1.94

		(1.34-6.88)		(0.41-3.88)		(0.02-5.25)		(0.00-9.11)		(1.00-3.38)
Aortic Aneurysm and Dissection	9	2.26# (1.03-4.28)	11	2.33# (1.16-4.16)	2	1.11 (0.13-4.00)	0	0.00 (0.00-5.48)	22	1.96# (1.23-2.97)
Other Diseases of Arteries, Arterioles, Capillaries	4	1.52 (0.41-3.89)	6	1.77 (0.65-3.86)	2	1.35 (0.16-4.87)	1	1.62 (0.04-9.02)	13	1.60 (0.85-2.74)
Pneumonia and Influenza	63	4.22# (3.25-5.40)	54	2.76# (2.07-3.60)	19	2.17# (1.31-3.39)	9	2.42# (1.11-4.60)	145	3.09# (2.61-3.63)
Chronic Obstructive Pulmonary Disease	97	2.23# (1.81-2.72)	146	2.58# (2.18-3.04)	72	2.88# (2.25-3.62)	36	3.41# (2.39-4.72)	351	2.59# (2.32-2.87)
Stomach and Duodenal Ulcers	1	1.05 (0.03-5.84)	4	3.41 (0.93-8.73)	1	2.10 (0.05-11.68)	0	0.00 (0.00-19.02)	6	2.14 (0.79-4.67)
Chronic Liver Disease and Cirrhosis	29	3.15# (2.11-4.53)	21	1.93# (1.20-2.95)	10	2.42# (1.16-4.44)	4	2.56 (0.70-6.54)	64	2.48# (1.91-3.17)
Nephritis, Nephrotic Syndrome and Nephrosis	27	2.01# (1.33-2.93)	18	1.02 (0.6-1.61)	8	0.99 (0.43-1.96)	7	2.06 (0.83-4.24)	60	1.41# (1.08-1.82)
Complications of Pregnancy,	1	230.94#	0	0.00	0	0.00	0	0.00	1	94.89#

Childbirth, Puerperium		(5.85-1,286.71)		(0.00-816.17)		(0.00-2,833.62)		(0.00-9,548.94)		(2.40-528.72)
Congenital Anomalies	0	0.00 (0.00-5.41)	1	1.23 (0.03-6.87)	1	3.22 (0.08-17.94)	0	0.00 (0.00-31.05)	2	1.04 (0.13-3.76)
Certain Conditions Originating in Perinatal Period	0	0.00 (0.00-1,154.17)	1	267.52# (6.77-1,490.53)	0	0.00 (0.00-2,627.65)	0	0.00 (0.00-6,998.12)	1	112.80# (2.86-628.49)
Symptoms, Signs and Ill-Defined Conditions	39	6.14# (4.36-8.39)	27	3.14# (2.07-4.57)	6	1.47 (0.54-3.20)	5	2.95 (0.96-6.89)	77	3.71# (2.93-4.64)
Accidents and Adverse Effects	31	1.47# (1.00-2.09)	44	1.67# (1.22-2.25)	20	1.78# (1.09-2.76)	7	1.47 (0.59-3.03)	102	1.61# (1.31-1.96)
Suicide and Self-Inflicted Injury	43	6.52# (4.72-8.78)	24	3.11# (1.99-4.63)	3	1.02 (0.21-2.98)	2	1.8 (0.22-6.50)	72	3.92# (3.07-4.94)
Homicide and Legal Intervention	0	0.00 (0.00-3.40)	5	4.39# (1.43-10.25)	0	0.00 (0.00-9.84)	0	0.00 (0.00-29.76)	5	1.84 (0.60-4.28)
Other Cause of Death	313	3.42# (3.05-3.82)	286	2.30# (2.04-2.58)	108	1.80# (1.48-2.17)	43	1.57# (1.14-2.12)	750	2.47# (2.30-2.65)

1 number of cancer patients who died due to each cause of death.

2 95% Confidence interval.

# P value less than .05.
